# Gait parameters of Parkinson’s disease compared with healthy controls: a systematic review and meta-analysis

**DOI:** 10.1038/s41598-020-80768-2

**Published:** 2021-01-12

**Authors:** Ana Paula Janner Zanardi, Edson Soares da Silva, Rochelle Rocha Costa, Elren Passos-Monteiro, Ivan Oliveira dos Santos, Luiz Fernando Martins Kruel, Leonardo Alexandre Peyré-Tartaruga

**Affiliations:** 1 Exercise Research Laboratory, Universidade Federal Do Rio Grande Do Sul, 750 Felizardo St, Porto Alegre, RS 90690-200 Brazil; 2 Univel University Center, Cascavel, Brazil; 3 Laboratory of PhysioMechanics of Locomotion, Universidade Federal Do Pará, Castanhal, Brazil

**Keywords:** Biomedical engineering, Skeletal muscle

## Abstract

We systematically reviewed observational and clinical trials (baseline) studies examining differences in gait parameters between Parkinson’s disease (PD) in on-medication state and healthy control. Four electronic databases were searched (November-2018 and updated in October-2020). Independent researchers identified studies that evaluated gait parameters measured quantitatively during self-selected walking speed. Risk of bias was assessed using an instrument proposed by Downs and Black (1998). Pooled effects were reported as standardized mean differences and 95% confidence intervals using a random-effects model. A total of 72 studies involving 3027 participants (1510 with PD and 1517 health control) met the inclusion criteria. The self-selected walking speed, stride length, swing time and hip excursion were reduced in people with PD compared with healthy control. Additionally, PD subjects presented higher cadence and double support time. Although with a smaller difference for treadmill, walking speed is reduced both on treadmill (.13 m s^−1^) and on overground (.17 m s^−1^) in PD. The self-select walking speed, stride length, cadence, double support, swing time and sagittal hip angle were altered in people with PD compared with healthy control. The precise determination of these modifications will be beneficial in determining which intervention elements are most critical in bringing about positive, clinically meaningful changes in individuals with PD (PROSPERO protocol CRD42018113042).

## Introduction

Parkinson’s disease (PD) is a chronic neurodegenerative condition characterized by decreased dopamine production in the substantia nigra pars compacta^1^. In addition, impairment in dopamine production resulting in changes on the cortical region is related to the planning and sequencing of the movements^2^.

In Brazil, the incidence of disease in 2005 was around 16 million, and the projection is that number can double in 2030^3^. Factors such as aging, male gender, and geographic location may be associated with differences in PD incidence. It has been demonstrated that prevalence is higher after 79 years in South American residents^4^. Although the causes for PD manifestation are unknown, some studies show an association with genetic and environmental factors^5,6^.

Some motor symptoms such as bradykinesia, postural instability, rest tremor, rigidity, and slowness of movement are present in PD^7^. These cardinal symptoms promote alteration in gait parameters in subjects with PD^8–10^. The literature has indicated that self-selected walking speed (SSWS) is reduced in people with PD^8,9^ when compared to a matched healthy control group^8^.

Notably, individuals with PD walk with higher cadence, shorter stride length, higher time in double limb support phase, greater asymmetry of upper and lower limbs, axial rigidity and reduced range of hip, knee and ankle motions^9–12^. The reduced range of motion (ROM) in lower limbs exerts influence on the short stride length at SSWS^10,12^. Further, PD motor disturbance is characterized by a reduction in walking speed due to a combination of reduced stride length and increased cadence^11,12^. Kinematic changes may relate to an inability to maintain proper joint excursions.

Although the literature indicates some gait characteristics in PD, several evaluation methods are applied, resulting in different reference values^9–11^. An extensive range of evaluation methods, disease duration, disease stages, phase of medication and aging process may hamper clarity over these biomechanical parameters, generating difficulty in proposing more effective rehabilitation programs.

A recent review showed the gait impairments in PD^13^, however, they aimed to study the assessment, mechanisms, and interventions to improve gait, and no metanalysis was performed. A systematic review and meta-analysis conducted by Creaby and Cole^14^ showed spatiotemporal and kinematic characteristics representing the risk of falls in individuals with PD^14^. The meta-analysis revealed that the likelihood of falls is higher in PD individuals presenting slower walking speed, lower cadence, shorter strides and more mediolateral head and pelvis motion. Still, spatiotemporal and kinematic analyses during walking compared with the healthy control group were not performed. No systematic reviews with meta-analysis were found comparing spatiotemporal and kinematic parameters while walking between PD and healthy control individuals. The quantitative characterization of gait parameters in individuals with PD might help researchers analyze this population data and help professionals observe PD's gait evolution after a rehabilitation program. Therefore, the aim of this study was to systematically review the literature about the spatiotemporal and lower limb angles during walking on people with PD compared with age-matched control subjects and perform meta-analyses. We hypothesized that the SSWS would be deteriorated (reduced speed), accompanied by a reduction in the stride length, swing time and lower limb angles, and higher cadence, step width and double support in individuals with PD compared with healthy controls.

## Methods

This systematic review has been reported according to the Guidelines for Meta-Analyses and Systematic Reviews of Observational Studies (MOOSE) (Supplementary material 1.1)^15^ and followed the recommendations proposed by the Preferred Reporting Items for Systematic review and Meta-Analysis Protocols (PRISMA-P) and Cochrane Collaboration^16^. The study protocol was pre-registered on the International Prospective Register of Systematic Reviews (PROSPERO protocol CRD42018113042). The present study has been published as a part of MSc dissertation of the first author (http://hdl.handle.net/10183/200663).

### Search strategy

The search was conducted in November 2018 and updated in October 2020 by two experienced investigators. The searching electronic bibliographic databases were Cochrane library, Scopus, Pubmed, and EMBASE. Abstracts or extended abstracts published from conferences, theses, dissertations, or studies not yet published in journals were not included. The following terms were used in combination and/or alone: ‘‘Parkinson disease,’’ “kinematics”, “joint kinematic”, “hip angles”, “knee angles”, “ankle angles”, “stride frequency”, “stride length”. Boolean operators ‘‘OR’’ and ‘‘AND’’ were used to search the databases. Details of the PubMed search are shown in Supplementary material 1.2. Also, a manual search of the reference lists of the studies found in the databases was conducted.

### Inclusion and exclusion criteria

This review included cross-sectional studies and clinical trials (from which only baseline values were extracted). To be considered eligible, studies should present: (1) straight-line free walking or treadmill walking evaluation with kinematic analysis; (2) people with PD as sample (evaluated in “on” period of medication, regardless of age, sex, and disease stage); (3) an age- and sex-matched healthy control group; (4) values (means and standard deviations) of spatiotemporal outcomes (during SSWS), walking distance, stride length, cadence, step width, double support, single support, swing time, sagittal hip, knee and ankle ROM throughout the gait cycle and at initial contact evaluated in SSWS. Some studies were excluded when (1) the variables of interest were not informed; (2) when subjects presented essential tremor, (3) postural alterations, such as camptocormia and Pisa syndrome; (4) de novo PD; (5) parkinsonism; (6) freezing and (7) differences speeds to both lower limbs; and (8) duplicate data. There were no restrictions on the date of publication for inclusion of studies in the review. Unpublished studies have not been included, and no hand searching was made. Only studies published in English, Portuguese, or Spanish were included. Excluded Studies are in Supplementary material 1.3.

### Selection of studies

The selection of studies was conducted independently by two reviewers (A.P.J.Z.; E.S.S.). First, titles and abstracts of studies found through the search strategy were evaluated considering the eligibility criteria. In the second phase, for the selected articles or those in doubt, the same two independent reviewers performed the full-text reading, and the eligibility criteria were followed. Disagreements between reviewers were resolved by consensus, and when necessary, by a third reviewer (R.R.C.).

Data extraction was performed by the same independently two reviewers who conducted the selection of studies. A standardized form containing the information of interest that should be extracted was delivered to each of the reviewers. The data extracted from the studies were: Age (years), mass (kg), height (m), Hoehn and Yahr scale (H&Y), score of the Unified Parkinson's disease Rating Scale (UPDRS), disease duration (years), type of walk test performed (free walking test or treadmill), SSWS (m s^−1^) and walking distance (m) (Supplementary material 1.4). In addition, means and standard deviations of the outcomes were extracted to the standardized form: SSWS (m s^−1^), walking distance (m), stride length (m), cadence (step min^−1^), step width (m), double support (%), single support (%), swing time (%), sagittal hip, knee and ankle ROM (°), ankle, hip, and knee angles (°) at initial contact evaluated in SSWS. The authors of the included studies were contacted by email, aiming to access possible unclear data. If no answer was received, the data in question were excluded from the analysis. In the results presented through figures (graphics), the software Image-J (National Institute of Health, USA) was used to achieve the outcome data. The justification for exclusion is in Supplementary material 1.3.

### Assessment of risk of bias (methodological quality)

A customized quality checklist was developed in this review, applying an instrument proposed by Downs and Black^17^. Other authors have been using this checklist with adequate and customized questions^18–20^. It was originally designed to assess the methodological quality of randomized and non-randomized studies of interventions. In this study, just observational studies were evaluated. Therefore, the instrument was developed by removing items 4, 8, 9, 13, 14, 15, 16, 17, 19, 23, 24, 25 and 26 because they were not relevant to these types of study. The included questions were 1, 2, 3, 5, 6, 7, 10, 11, 12, 18, 20, 21 and 22. The computation of quality of studies was based on Ratcliffe and collaborators^21^, studies scored as high quality achieve a score > 66.8%, medium quality 33.4–66.7%, and low-quality studies achieving < 33.3%.

### Data analysis

The pooled effect estimates were computed from the difference scores between the gait parameters of PD individuals and the healthy controls, their standard deviations, and the number of participants. The authors were contacted through emails for unreported data and, if no answer was returned or the data requested were not available, the studies were excluded. To be included in the meta-analysis study needed to present the mean, standard deviation, standard error, or upper and lower limits of 95% confidence interval.

The results of meta-analyses are exhibited as standardized mean differences and calculations were performed using random-effects models. Statistical heterogeneity of evaluations among studies was evaluated by Cochran’s Q test and the I^2^ inconsistency test; it was considered that values > 50% indicated high heterogeneity^16^. Also, sensitivity analyses were conducted to investigate the possible influence of the method selected to assess gait parameters in the included studies on the differences between PD and healthy people, separating the studies using free walking of those using treadmill. Meta-regression analyses were performed to investigate potential moderators: mean age (years), mean H&Y (scores), mean UPDRS (scores) and mean disease duration (years). For descriptive analysis, the mean and standard deviation of each outcome in each group was presented (Supplementary material 2.6). Subgroup (treadmill versus free walking) and meta-regression analyses were not performed for joint kinematic from lower limbs (ROM and angle at initial contact) because there were not enough studies. Also, subgroup analyses were not performed for stride length, step width, double support time, single support time, swing time because there were not enough studies. Furthermore, publication bias was assessed using funnel plots for each outcome (of each trial's effect size against the standard error). Funnel plot asymmetry was evaluated using Begg and Egger tests^22^ and significant publication bias was considered if the *p* value < 0.05. Trim-and-fill computation was used to estimate the effect of publication bias on the interpretation of results^23^.

Forest plots were generated indicating the pooled effects and standardized mean differences, with 95% confidence intervals (CIs) for each outcome. Values of *p* < 0.05 were considered statistically significant. All analyses were performed using Comprehensive Meta-Analysis software version 3.3.07. All individual data are in Supplementary material 2.7.

## Results

### Studies selection

**Figure 1:**
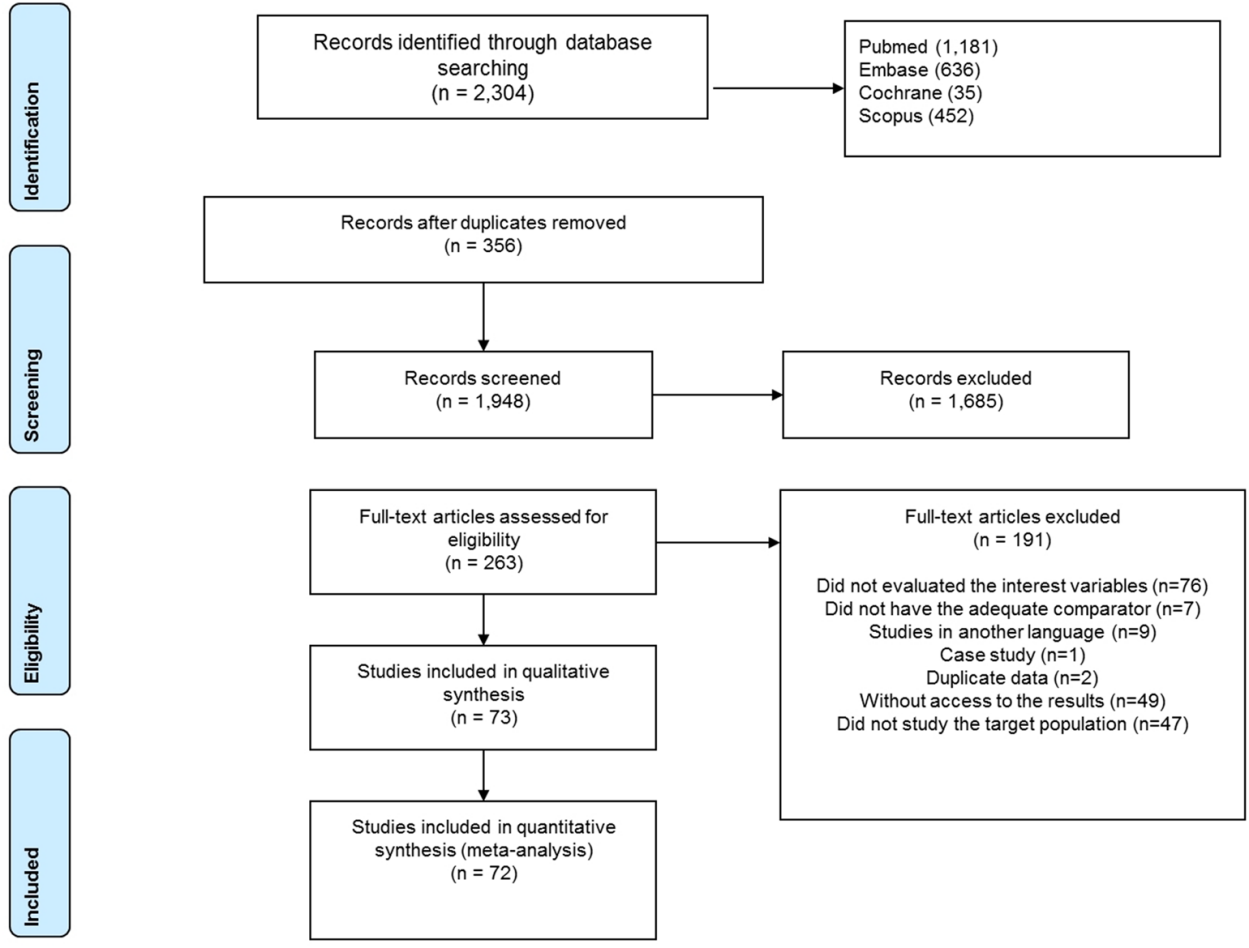
Flowchart of number of articles retrieved during the literature search and study selection.

A total of 2398 studies were identified during the literature search. After adjusting for duplicates, 2042 studies remained. No additional articles were included in the present review, resulting from the manual search performed. After reading the abstracts, 1775 were removed, as they did not contain the key concepts of the study question. The full texts of 267 studies were read, and, from this analysis, 194 studies were excluded. Most of these studies were excluded either because (1) the study did not evaluate gait variables, (2) evaluation performed in OFF medication, (3) lack of control group, (4) post-deep brain stimulation (DBS) surgery (5) characteristic of a preliminary study. Thus, 72 studies met the inclusion criteria and were included in the quantitative analysis (Fig. 1). Three trials were included twice because they had met the eligibility criteria for two comparison groups. No other search was performed.

### Characteristics of the included studies

In total, 72 studies and 76 comparison pairs were found. In this review, 3027 participants were included in the meta-analyses. Among these, 1510 and 1517 participants were from PD groups and control groups, respectively. 77% of the studies showed H&Y values, 66% provide UPDRS information, 62% of the studies exhibited the disease duration, 100% informed age of PD group, and 1% did not provide this information about control group. The characteristics of the 72 included studies are available in Supplementary material 1.4.

### Methodological quality of the included trials

Of the 72 included studies, 100% showed the hypothesis/aim/objective clearly described, 97% described the primary outcomes, 59% indicated the characteristics of participants clearly, 99% described principal confounders, 100% reported the main findings, 100% showed random variability in the data, 82% described probability values, in 75% of the studies the participants are representative of the population, 93% measured the appropriate statistic, 100% measured the main outcome if accurate methods, 100% recruited the participants of the same population and 8% of the studies recruited the participants of the groups in the same period (Supplementary material 1.5).

### Gait Parameters of PD and healthy

#### Meta-analysis of speed

**Figure 2:**
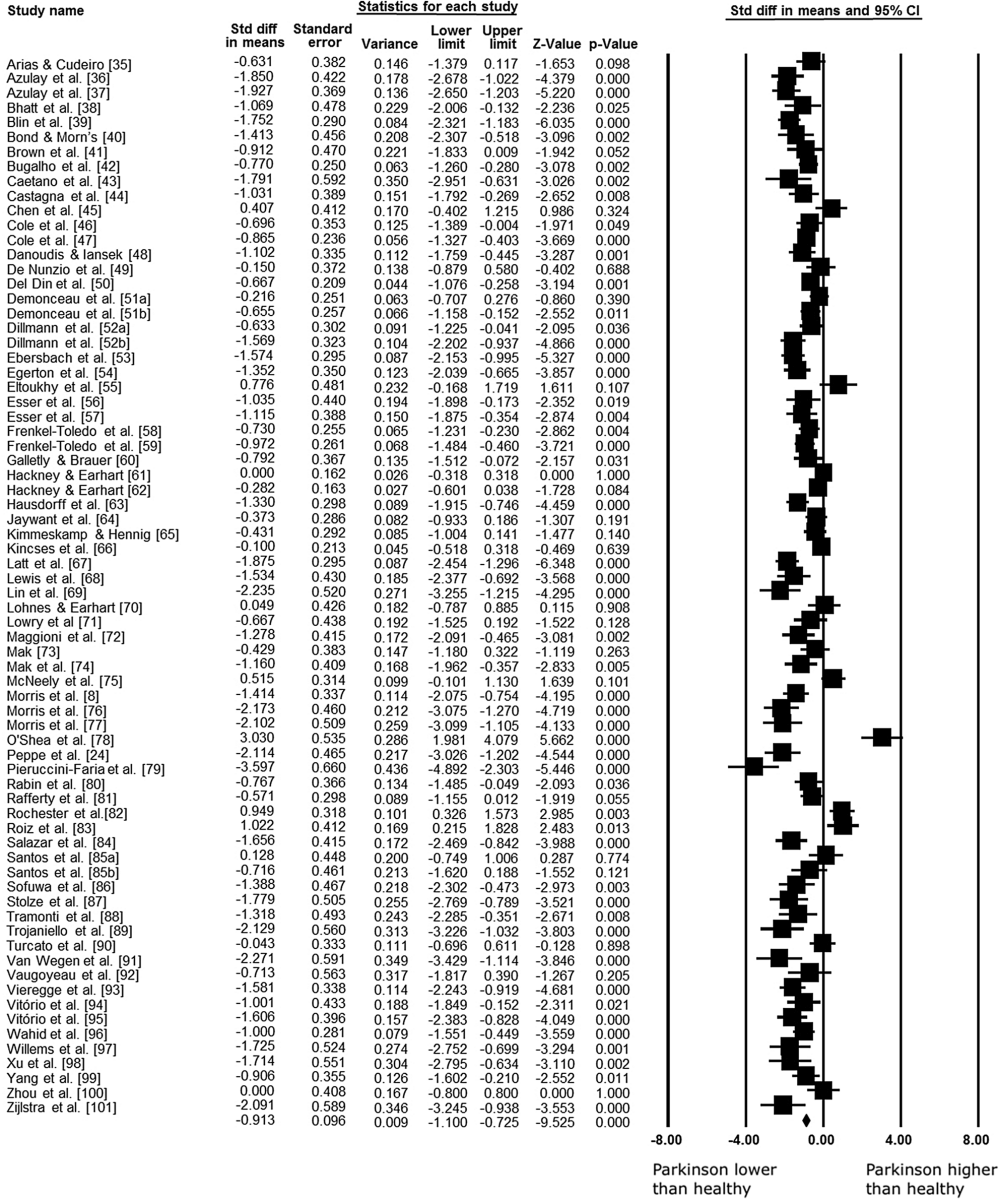
Standardized mean differences on gait speed between Parkinson and healthy individuals. *CI* confidence interval, *Std diff* standardized difference.

Data concerning speed were available from 69 studies and 72 combination pairs, which compare the speed of PD versus healthy group, in 2932 participants. Meta-analysis showed that speed is approximately 0.17 m s^−1^ reduced in people with PD compared with healthy group (ES: − 0.913; 95% CI − 1.100 to − 0.725; *p* < 0.001; I^2^: 81%) (Fig. 2). However, the analysis of publication bias for this outcome identified a significant bias (*p* = 0.003), and thus the adjusted value of the effect size, according to the Duval and Tweedie’s trim and fill test, resulted in − 0.619 (95% CI − 0.809 to − 0.429).

Subgroup analysis of studies showed that PD participants walked slower than healthy controls both during free (66 studies; 68 combination pairs; ES: − 0.914; 95% CI − 1.113 to − 0.716; *p* < 0.001; I^2^: 82%; − 0.17 m s^−1^) and treadmill walking (3 studies; 4 combination pairs; ES: − 0.919; 95% CI − 1.376 to − 0.462; *p* < 0.001; I^2^: 54%; − 0.13 m s^−1^). More information on these studies is in Supplementary material 1.4. According to the results of meta-regression analysis, mean age, H&Y, UPDRS, and disease duration did not influence the gait speed difference between PD versus the healthy control group (Table 1).

**Table 1:** Meta-regression of moderators of the gait parameters of Parkinson’s disease.

Outcome/moderator	Number of study estimates	β	95% CI	*p* value	R^2^
**Speed**					
Age	26	0.053	0.033 to 0.140	0.227	0.01
H&Y	26	0.124	0.546 to 0.814	0.722	− 0.04
UPDRS	26	0.013	0.045 to 0.018	0.414	− 0.05
Disease duration	26	0.020	0.131 to 0.173	0.789	− 0.05
**Stride length**					
Age	21	− 0.001	− 0.080 to 0.080	0.997	− 0.11
H&Y	21	− 0.586	− 1.283 to 0.110	0.098	0.15
UPDRS	21	− 0.022	− 0.061 to 0.015	0.244	0.00
Disease duration	21	0.003	− 0.146 to 0.152	0.960	− 0.10
**Cadence**					
Age	16	0.041	0.137 to 0.055	0.400	− 0.05
H&Y	16	0.558	0.191 to 0.075	0.084	0.23
UPDRS	16	0.003	0.026 to 0.032	0.840	− 0.08
Disease duration	16	0.061	0.124 to 0.247	0.515	− 0.05
**Step width**					
Age	7	0.230	0.121 to 0.469	0.058	0.37
H&Y	7	0.371	0.779 to 1.522	0.526	− 0.54
UPDRS	7	0.005	0.264 to 0.253	0.967	− 0.31
Disease duration	7	0.106	0.233 to 0.447	0.539	− 0.29
**Double support**					
Age	7	0.001	0.183 to 0.179	0.986	− 0.32
H&Y	7	0.170	0.469 to 1.811	0.838	− 0.31
UPDRS	7	0.006	0.107 to 0.120	0.913	− 0.31
Disease duration	7	0.026	0.053 to 0.001	0.058	0.49
**Single support**					
Age	4	0.163	0.836 to 0.509	0.633	− 0.40
H&Y	4	0.940	2.887 to 4.768	0.630	− 0.37
UPDRS	4	0.020	0.173 to 0.132	0.794	− 0.46
Disease duration	4	0.134	0.340 to 0.609	0.579	− 0.40
**Swing time**					
Age	11	0.069	0.226 to 0.086	0.381	− 0.12
H&Y	4	2.535	0.083 to 4.987	0.**042**	0.57
UPDRS	6	0.149	0.246 to 0.052	0.**002**	0.67
Disease duration	5	0.177	0.115 to 0.469	0.234	0.06

*p*-values in bold indicate statistical significance (*p* < 0.05).

*H&Y* Hoehn and Yahr scale, *UPDRS* unified Parkinson's disease rating scale.

#### Meta-analysis of stride length

**Figure 3:**
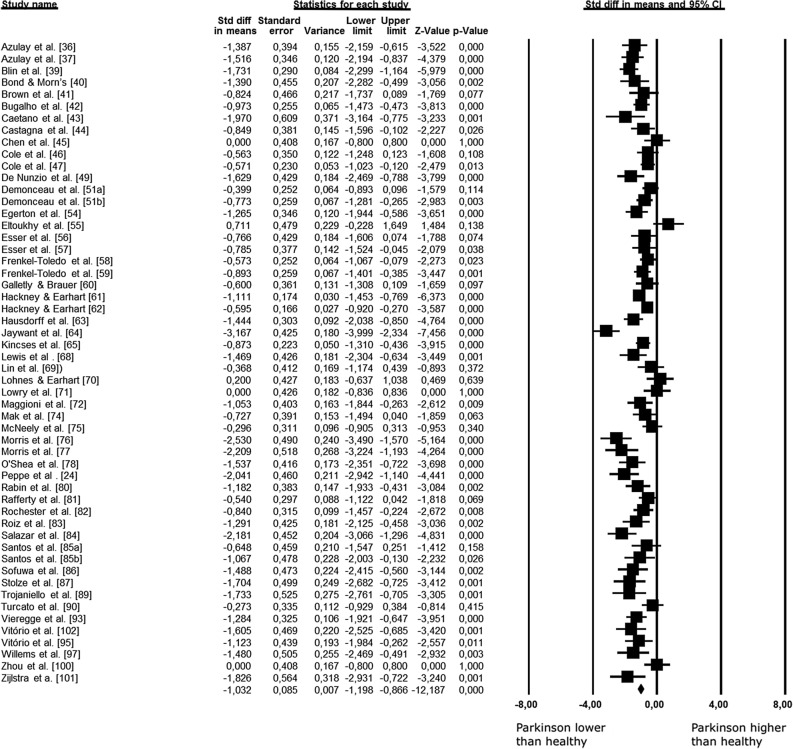
Standardized mean differences on stride length between Parkinson and healthy individuals. *CI* confidence interval, *Std diff* standardized difference.

Data concerning stride length were available from 52 studies and 54 combination pairs, which compare stride length of PD versus healthy group, in a total of 2188 participants. Meta-analysis demonstrated that stride length is approximately 0.16 m reduced in PD compared with healthy groups (ES: − 1.032; 95% CI − 1.198 to − 0.866; *p* < 0.001; I^2^: 67%) (Fig. 3). However, the analysis of publication bias for this outcome identified a significant bias (*p* = 0.003), and thus the adjusted value of the effect size, according to the Duval and Tweedie’s trim and fill test, resulted in − 0.836 (95% CI − 1.017 to − 0.655). The meta-regression analysis showed that mean age, H&Y, UPDRS, and disease duration did not influence the stride length difference between individuals with PD and healthy controls (Table 1).

#### Meta-analysis of cadence

**Figure 4:**
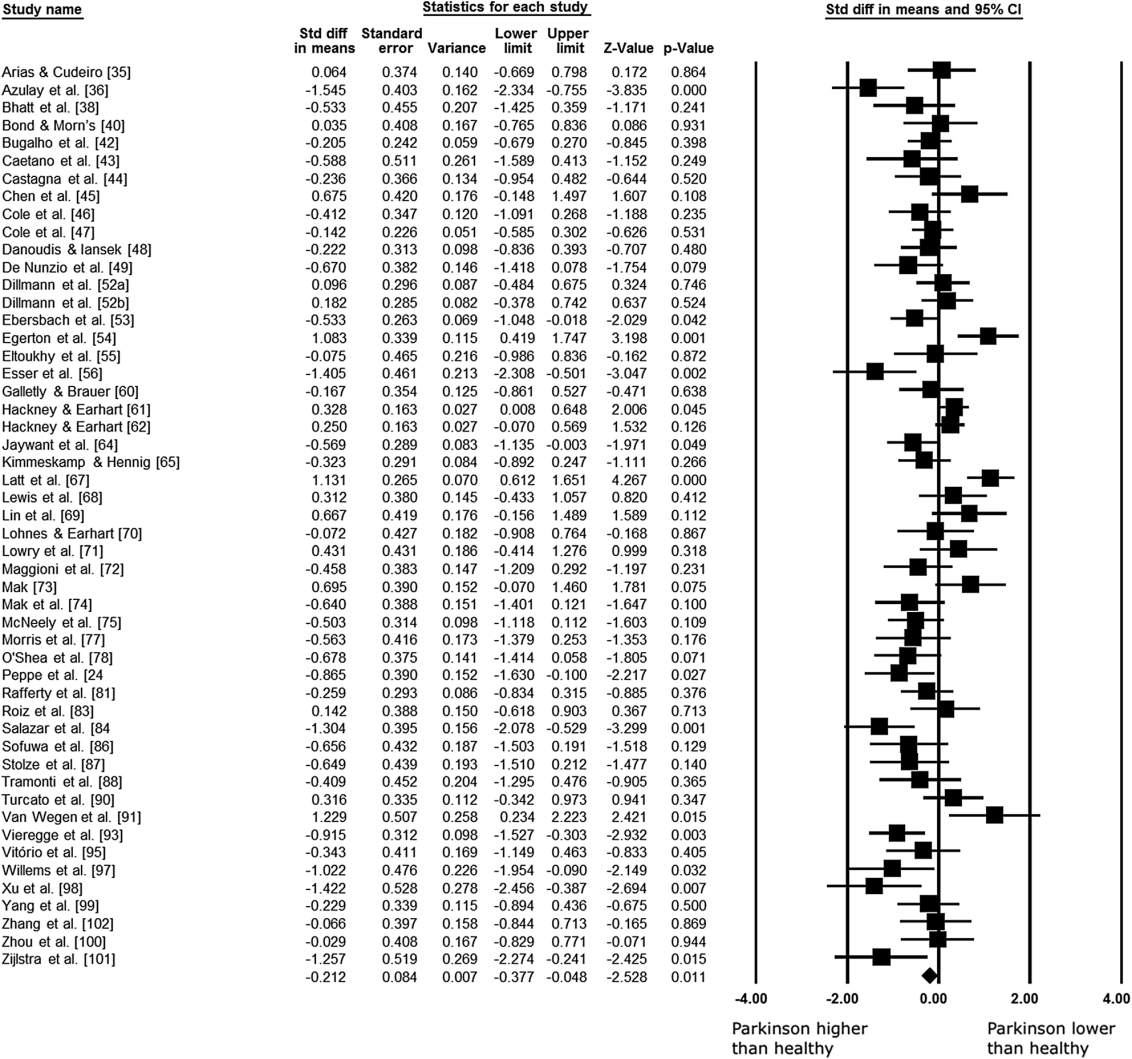
Standardized mean differences on gait cadence between Parkinson and healthy individuals. *CI* confidence interval, *Std diff* standardized difference.

Data concerning cadence were available from 50 studies and 51 combination pairs, which compare cadence of PD versus healthy group, in a total of 1936 participants. Meta-analysis showed that cadence is approximately 1.75 step min^−1^ higher in PD compared with healthy groups (ES: − 0.212; 95% CI − 0.377 to − 0.048; *p* = 0.011; I^2^: 66%) (Fig. 4). The analysis of publication bias for this outcome showed no significant bias (*p* = 0.074).

Subgroup analysis of studies, which evaluated cadence using free (ground) walking or treadmill, evidenced that this criterion influences the gait differences between PD and healthy groups. Studies adopting free walking strategy to evaluate this variable demonstrated that cadence is 1.86 step min^−1^ higher in PD subjects compared to healthy groups (ES: − 0.228; 95% CI − 0.402 to − 0.054; *p* < 0.001; I^2^: 67%). In contrast, studies using the treadmill to evaluate the cadence (3 studies) did not show a difference between the cadence of PD and healthy groups (ES: − 0.023; 95% CI − 0.550 to 0.503; *p* = 0.931; I^2^: 52%). Furthermore, the meta-regression analysis results demonstrated that mean age, H&Y, UPDRS, and disease duration do not influence the cadence difference between PD subjects and healthy groups (Table 1).

#### Meta-analysis of step width

Data concerning step width were available from 18 studies and 19 combination pairs, which compare the step width of PD versus healthy group, in 628 participants. Meta-analysis demonstrated that step width did not differ between PD and healthy groups (ES: 0.104; 95% CI − 0.153 to 0.361; *p* = 0.426; I^2^: 59%) (Supplementary material 1.6) The analysis of publication bias for this outcome showed no significant bias (*p* = 0.327). Besides, the meta-regression analysis showed that mean age, H&Y, UPDRS, and disease duration do not influence the step width difference between individuals with PD and healthy controls (Table 1).

#### Meta-analysis of double support time

Data concerning double support time were available from 15 studies and 16 combination pairs, which compared the double support time of PD versus control groups, in a total of 562 participants. Meta-analysis showed that double support time is approximately 1.79% longer in PD compared with healthy groups (ES: 0.489; 95% CI 0.137 to 0.841; *p* < 0.001; I^2^: 73%) (Supplementary material 1.7). The analysis of publication bias for this outcome showed no significant bias (*p* = 0.260). The meta-regression analysis showed that mean age, H&Y, UPDRS, and disease duration do not influence the double support time difference between PD subjects and healthy groups (Table 1).

#### Meta-analysis of single support time

Data concerning single support were available from 10 studies, which compared single support time between individuals with PD versus healthy group, in a total of 366 participants. Meta-analysis demonstrated that single support time did not differ between PD and healthy groups (ES: 0.273; 95% CI − 0.204 to 0.750; *p* = 0.262; I^2^: 83%) (Supplementary material 1.8). The analysis of publication bias for this outcome showed no significant bias (*p* = 0.720). In addition, the meta-regression analysis showed that mean age, H&Y, UPDRS, and disease duration do not influence the single support time difference between PD subjects and healthy groups (Table 1).

#### Meta-analysis of swing time

Data concerning swing phase time were available from 11 studies, which compare the swing time of PD versus healthy group, in 661 participants. Meta-analysis showed that swing time is 1.76% reduced in PD compared with healthy groups (ES: − 0.715; 95% CI − 1.096 to − 0.334; *p* < 0.001; I^2^: 79%) (Supplementary material 1.9). The analysis of publication bias for this outcome showed no significant bias (*p* = 0.087).

According to the results of meta-regression analysis, mean age and disease duration do not influence the swing time difference between PD subjects and healthy groups. On the other hand, the disease stage evaluated by H&Y plays a significant role in the swing phase time difference between PD and healthy groups (β: 2.535; 95% CI 0.084–4.987 *p* = 0.042; R^2^ = 0.57). Therefore, the higher the H&Y values, the higher is the difference between swing phase time in PD compared with healthy groups. Conversely, the UPDRS exert a significant influence on the swing phase difference between PD and healthy groups (β: − 0.149; 95% CI − 0.247 to − 0.052 *p* = 0.002; R^2^ = 0.67). The difference between PD and healthy groups decreased with increasing severity of PD (Table 1).

#### Meta-analysis of hip ROM

Data concerning hip ROM were available from 3 studies, which compare hip ROM of PD versus healthy group in 76 participants. Meta-analysis demonstrated that hip ROM is 5.29 degrees reduced in PD compared with healthy groups (ES: − 0.860; 95% CI − 1.333 to − 0.388 *p* < 0.001; I^2^: 0%) (Supplementary material 2.0).

#### Meta-analysis of knee ROM

Data concerning knee ROM were available from 2 studies, which compare knee ROM of PD versus healthy groups in 52 participants. Meta-analysis showed that knee ROM did not differ between PD and healthy groups (ES: − 1.033; 95% CI − 2.564 to 0.498; *p* = 0.186; I^2^: 82%) (Supplementary material 2.1).

#### Meta-analysis of ankle ROM

Data concerning ankle ROM were available from 3 studies, which compare ankle ROM of PD versus control groups, in a total of 76 participants. Meta-analysis demonstrated that ankle ROM did not differ between PD and healthy groups (ES: − 0.216; 95% CI − 0.896 to 465; *p* = 0.534; I^2^: 53%) (Supplementary material 2.2).

#### Meta-analysis of hip angle at initial contact

Data concerning hip angle at initial contact were available from 3 studies, which compared hip angle at initial contact of PD versus control groups in a total of 70 participants. Meta-analysis demonstrated that hip angle at initial contact did not differ between PD and healthy groups (ES: − 1.023; 95% CI − 2.291 to 0.245; *p* = 0.114; I^2^: 83%) (Supplementary material 2.3).

#### Meta-analysis of knee angle at initial contact

Data concerning knee angle at initial contact were available from 3 studies, which compared the knee angle at initial contact of PD versus healthy group, in a total of 70 participants. Meta-analysis showed that knee angle at initial contact did not differ between PD and healthy groups (ES: 0.210; 95% CI − 0.395 to 0.814; *p* = 0.496; I^2^: 35%) (Supplementary material 2.4).

#### Meta-analysis of ankle angle at initial contact

Data concerning ankle angle at initial contact were available from 3 studies, which compared the ankle angle at initial contact of PD versus control groups in a total of 70 participants. Meta-analysis showed that the ankle angle at initial contact did not differ between PD and healthy groups (ES: 0.188; 95% CI − 0.290 to 0.666; *p* = 0.440; I^2^: 0%) (Supplementary material 2.5).

## Discussion

To the best of our knowledge, this is the first meta-analysis of published studies regarding the spatiotemporal and lower limb angles during SSWS on people with PD compared with healthy control subjects. The main results agree with our hypotheses showing that SSWS, stride length, cadence, double support, swing time and sagittal hip angle were different in people with PD compared with healthy control participants. In some cases, the method of evaluation of walk can influence these variables. Furthermore, the present study strongly contributes to the literature regarding PD gait characteristics, addressing measures that were not yet elucidated, such as (1) speed is 0.17 m s^−1^ reduced, (2) stride length is 0.16 m reduced, (3) cadence is 1.75 step min^−1^ higher; (4) double support time is 1.79% longer, (5) swing time is 1.76% reduced, and (vi) hip sagittal ROM is 5 degrees reduced in people with PD compared with the healthy control group.

### Spatiotemporal variables

Walking speed is an essential parameter of functional activities in daily life. Also, the walking speed test is a practical method useful for monitoring people's mobility with PD^24^. In this review, using 69 studies, we observed that PD SSWS is 0.17 m s^−1^ slower than healthy control group. Possibly, the bradykinesia and rigidity associated with physical inactivity may be a contributing factor, but the studies did not measure these outcomes^25^. The slower walking speeds are associated with mortality, hospitalization, frailty, and risk of falling^24^. Creaby and Cole^14^ revealed that reduced walking speeds denote a compensation strategy to avoid fallings, causing alterations, especially in spatiotemporal variables in individuals with PD^13^.

Gait speed is strictly related to stride length and cadence. Our systematic review with metanalysis showed that individuals with PD walk slower than healthy controls through a largely reduced stride length (0.16 m) despite showing higher cadence. Therefore, the relation stride length versus cadence is altered in PD which disagrees with previous findings that found this relationship unaltered^26^. Importantly, the cadence is similar between PD and healthy control during walking on the treadmill. The similarity is probably due to imposed treadmill speed (less-ecological task). These different results due to walking conditions need to be considered during rehabilitation interventions. One consequence of these changes is the reduced external mechanical work, mainly due to shorter stride length and reduced hip excursion^10^.

The findings of double support and swing time may be associated with gait instability in people with PD. In Peppe and collaborators’ study^25^, the long double support time was attributed to an inability to transfer weight in preparation for stepping adequately. In addition, swing time was reduced in people with PD compared with healthy control group, probably due to reduced walking speed, reduced stride length and higher cadence and double support time, resulting in reduced dynamical stability of gait in PD. Further, we found that level of disease progression affected the swing time. While the general scale of disease progression (H&Y) shows an increase in differences for swing time between PD individuals and healthy controls according to the severity of motor symptoms, the motor scale of disease progression (UPDRS) surprisingly showed a reduction of these differences according to the disease severity. This intriguing finding needs further consideration in future studies to understand the causes of this discrepancy. It has been suggested to include more sensitive measurements to associate the PD stage and their consequences on gait pattern^27^. Although double support time and swing time have been impaired, the step width remains unchanged, showing that lateral margins of stability are maintained in PD. These temporal alterations of gait are considered strategies of the neural system to reduce the risk of falls, allowing an enhanced postural control^28^.

The walking speed was reduced between individuals with PD and without PD in both free and treadmill walking conditions. Despite the reduction was 0.17 m s^−1^ in overground and 0.13 m s^−1^ in treadmill, both conditions can detect differences in that important marker of functionality. In general, the SSWS is reduced in treadmill compared with overground because most protocols using treadmill does not allow people to select their speed initially but instead choose one for them and then let them go up down from there^29^.

Mostly, gait alterations in people with PD occur at the early disease stage, evolving from uni to bilateral alteration^13^. The participants evaluated from the studies were somewhat homogeneous and, therefore, resulting in poor relation between disease stage and gait performance. Future studies in this field should include advanced staging and young PD as well as analysis with ON and OFF state of medication^13^.

### Angular variables

In addition to the spatiotemporal variables, angular measurements are relevant to characterize the walking pattern. The pelvic rotation, tilt and lateral oscillation, knee flexion in stance phase, foot on heel-strike and toe-off are determinants to recovery energy and avoid compensations during walking^30^. The present study showed that only the range of hip motion was reduced (5°) during SSWS for individuals with PD compared to controls, while the knee and ankle ROM remained unaltered. The reduced hip excursion is probably accompanied by a reduced knee extension in the terminal stance phase^10^. Additionally, there is a reduced activity of *gastrocnemius medial* and higher activity of *tibialis anterior*, accompanied by a higher co-contraction of these ankle muscles during gait^12^. These changes influence adequately transfer weight in preparation for stepping and it can reflect in a higher metabolic cost of walking^10,12,30^.

No differences in knee and ankle sagittal ROM and hip, knee and ankle angles at initial contact were found between people with PD compared with healthy control group. However, DiPaola et al.^10^ found that knee ROM is critical, influencing the pendular mechanism of walking. Few studies analyzed these variables, therefore precluding the meta-regression analysis. The walking parameters in individuals with PD may be improved, and the variables that showed alterations in the present study should be the focus of rehabilitation and exercise interventions^31,32^. For example, some interventions like dance and Nordic walking have the potential to improve gait biomechanics and energetics in people with PD. These exercise modalities combine auditory stimulus, rhythmicity with direction changes, and large joint excursions^33,34^.

A significant contribution of the present analysis to the literature is comparing gait between people with relatively low disease severity in ON state of medication and healthy control group, which showed quantitatively how the variables differ from people with PD and healthy group. It was possible to affirm that SSWS, stride length, swing time, hip sagittal ROM are reduced and cadence and double support are higher during gait in people with PD. These findings can support health professionals to monitor the interventions to improve the gait parameters.

Finally, we highlight the first systematic review with sensitivity analysis and meta-regression that measured the differences in the gait of people with PD compared with healthy control groups. The high heterogeneity of some comparisons is a limitation of the present study. However, in general, the studies showed high methodological quality. Besides, more original studies are needed to explore the possible alterations in angular parameters. Our findings using treadmill protocols to evaluate gait biomechanics in PD individuals are preliminary, and further research is required to address this issue. This review selected studies with the ON phase of medication because these populations usually do the daily life in this phase of medication. Thus, more investigations are needed to explore the role of medication on gait.

## Conclusion

The present meta-analysis showed that people with PD have differences in gait characteristics compared with healthy control group. Different evaluation methods can influence some biomechanical parameters, though the PD’s main alterations are sensible in overground and treadmill setups. Based on our results, the subjects were homogeneous and meta-regression analysis showed that age, disease duration, H&Y and UPDRS, in general, did not exert influence over walking biomechanics.

## Supplementary Information

Supplementary Information 1.1–1.5.

Supplementary Information 1.6.

Supplementary Information 1.7.

Supplementary Information 1.8.

Supplementary Information 1.9.

Supplementary Information 2.0.

Supplementary Information 2.1.

Supplementary Information 2.2.

Supplementary Information 2.3.

Supplementary Information 2.4.

Supplementary Information 2.5.

Supplementary Information 2.6.

Supplementary Information 2.7.
