# Supplementary material for: Gait parameters of Parkinson’s disease compared with healthy controls: a systematic review and meta-analysis
Source: Sci Rep. 2021 Jan 12;11:752. doi: 10.1038/s41598-020-80768-2 (PMC7804291; doi:10.1038/s41598-020-80768-2)
Supplement: Supplementary file 1 — Supplementary Information 1.1–1.5. [file 41598_2020_80768_MOESM1_ESM.docx]

Supplementary material 1.1


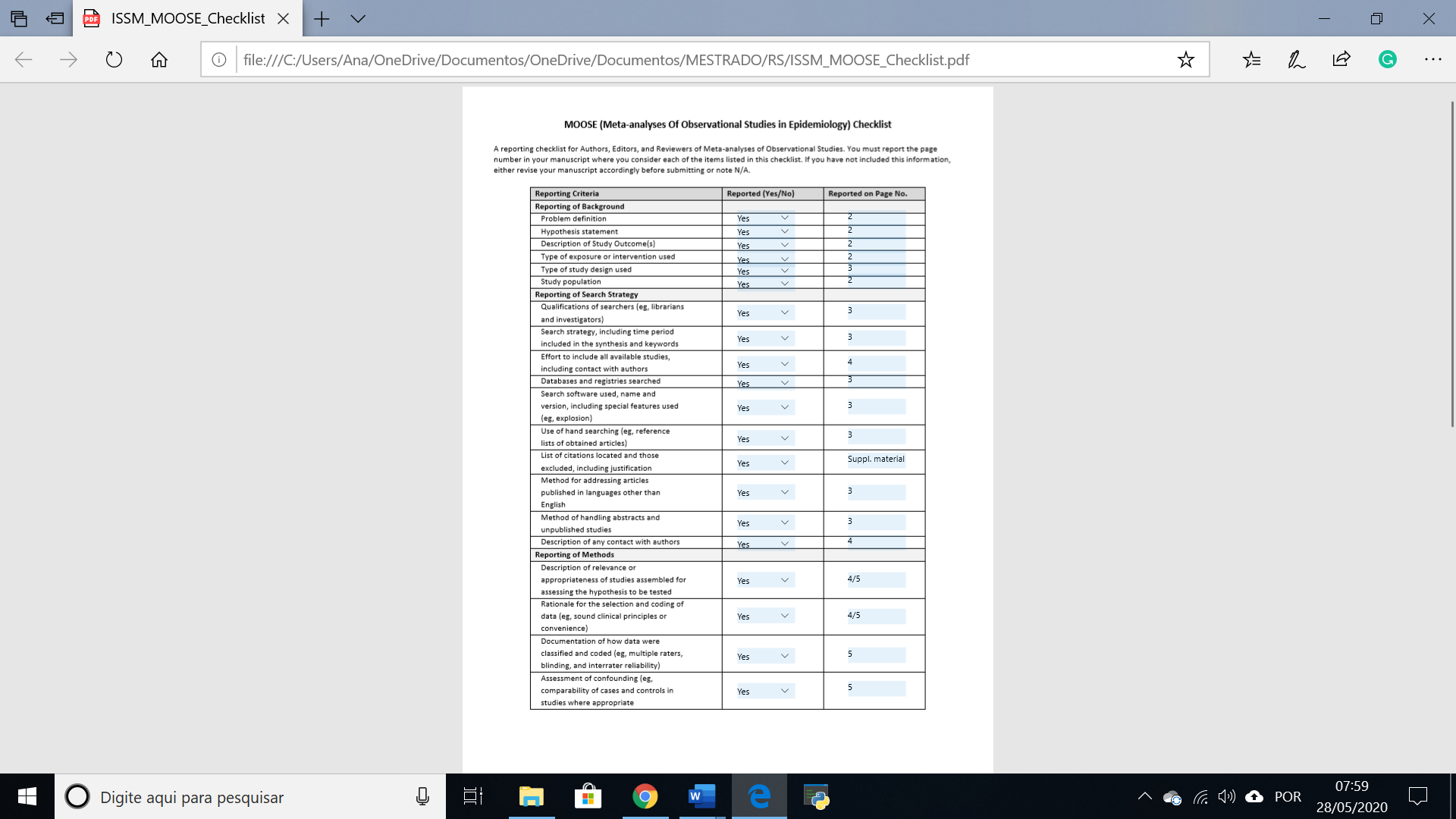


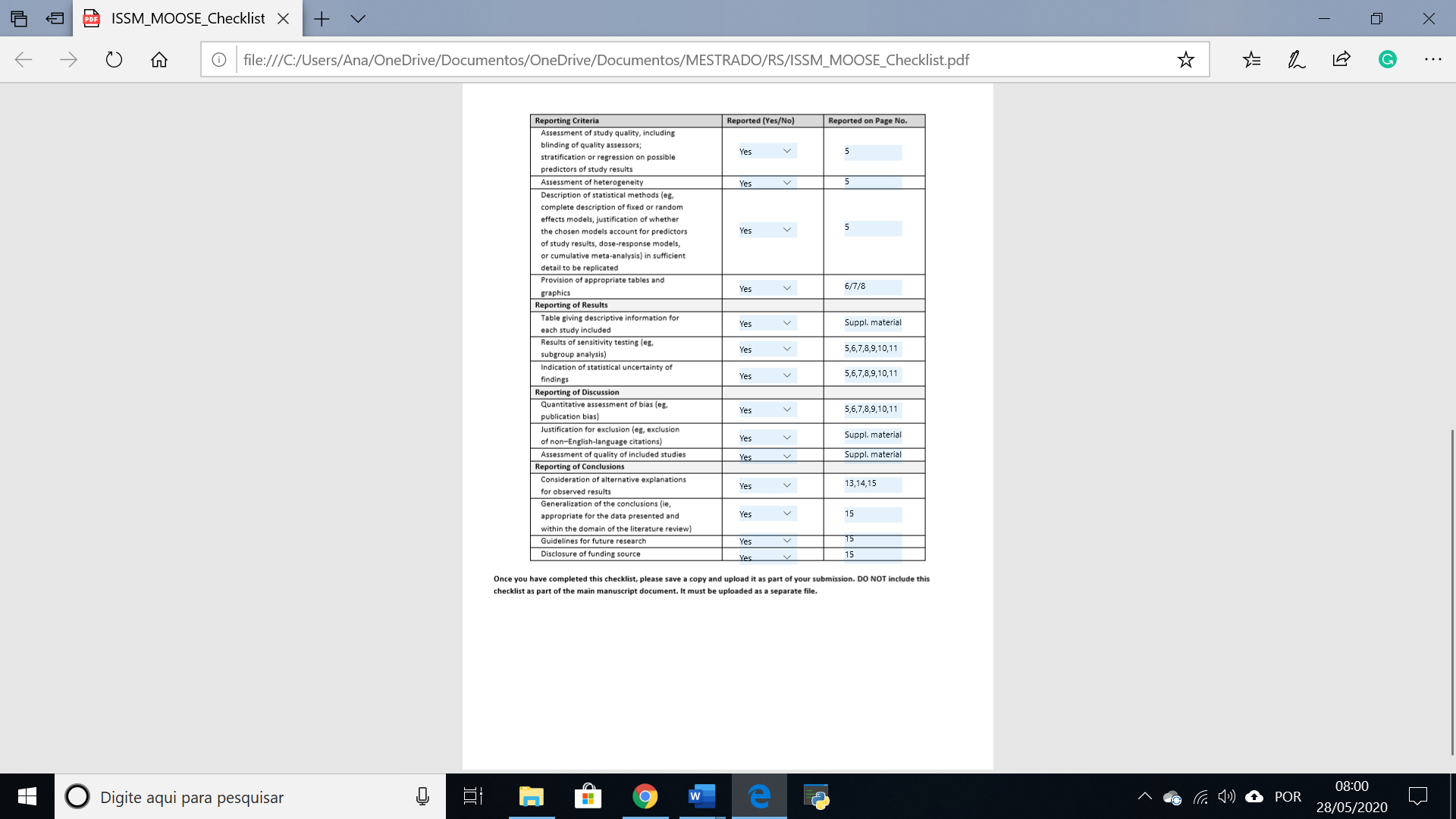


Supplementary material 1.2

PubMed search

Parkinson Disease"[Mesh] OR "Parkinson Disease" OR "Idiopathic Parkinson's Disease" OR "Lewy Body Parkinson Disease" OR "Lewy Body Parkinson's Disease" OR "Primary Parkinsonism" OR "Parkinsonism, Primary" OR "Parkinson Disease, Idiopathic" OR "Parkinson's Disease" OR "Parkinson's Disease" OR "Parkinson's Disease, Idiopathic" OR "Parkinson's Disease, Lewy Body" OR "Idiopathic Parkinson Disease" OR "Paralysis Agitans")) AND (Kinematic OR "joint kinematic" OR "hip angles" OR "knee angles" OR "ankle angles" OR "stride frequency" OR "length of stride")

Supplementary material 1.3

Excluded Studies

| Study | EXCLUSION JUSTIFICATION |
| --- | --- |
| Albani et al.(2016) | No variables |
| Andrew (2002) | No full text |
| Afsar et al.(2016) | No variables |
| Agosti et al.(2016) | No variables |
| Albani et al.(2012) | No variables |
| Allert et al.(2001) | OFF medication |
| Almeida et al.(2007) | No variables |
| Auvinet et al.(2014) | No variables |
| Azulay et al.(1996) | No full text |
| Barbieri et al.(2016) | No variables |
| Barbieri et al.(2018) | No variables |
| Bayle et al.(2016) | No variables |
| Beaulieu et al.(2018) | off medication |
| Bekkers et al.(2017) | No variables |
| Bello et al.(2008) | OFF medication |
| Bertoli et al.(2018) | No variables |
| Beuter et al.(1992) | No variables |
| Bjarnason et al.(2005) | No control group |
| Blin et al.(1990) | Pilot study |
| Brodie et al.(2015) | Pilot study |
| Bryant et al.(2015) | No control group |
| Buckley et al.(2008) | No variables |
| Cao et al.(2017) | No variables |
| Calabrò et al. (2019) | No variables |
| Capato et al.(2012) | No variables |
| Carpinella et al.(2007) | Post DBS |
| Castagna et al.(2012) | No variables |
| Castagna et al.(2013) | No variables |
| Cikajlo and Potisk (2019) | No variables |
| Chastan et al.(2009) | No variables |
| Chawla et al.(2014) | No control group |
| Chee et al.(2009) | OFF medication |
| Cho et al.(2010) | No variables |
| Cole et al.(2011) | Duplicate data |
| Conradsson et al.(2017) | No variables |
| Costa-Ribeiro et al.(2017) | Pilot study |
| Cowie et al.(2012) | Post DBS |
| Crenna et al.(2007) | No variables |
| Crenna et al.(2008) | Post DBS |
| De Aguiar Yamada et al.(2016) | No control group |
| Delval et al.(2006) | No variables |
| Delval et al.(2008) | OFF medication |
| Delval et al.(2010) | OFF medication |
| Dibble et al.(2004) | No variables |
| Dietz et al.(1995) | No variables |
| Dipaola et al.(2016) | OFF medication |
| Doan et al.(2013) | No variables |
| Djuric-Jovicic et al.(2017) | De novo subjects |
| Ehgoetz Martens et al.(2015) | No variables |
| Ewenczyk et al., 2017 | No variables |
| Faist et al.(2001) | Post DBS |
| Fernandez-del-Olmo & Sanchez (2015) | Letter |
| Ferrarin et al.(2002) | Pilot study |
| Ferrarin et al.(2004) | piloto |
| Ferrarin et al.(2006) | No variables |
| Fino et al.(2018) | OFF medication |
| Galli et al.(2018) | Pilot study |
| Galna et al.(2013) | No variables |
| Gilmore et al.(2015) | Post DBS |
| Gigot et al.(2016) | No variables |
| Ginis et al.(2017) | Pilot study |
| Grajic et al.(2015) | De novo |
| Halliday et al.(1998) | No variables |
| Hanakawa et al.(1999) | OFF medication |
| Harrison et al.(2018) | No control group |
| Hatanaka et al.(2016) | No variables |
| Horak et al.(2016) | No variables |
| Huang et al.(2012) | No variables |
| Hundza et al.(2014) | No variables |
| Huxham et al.(2008) | No variables |
| Jeon et al.(2008) | No variables |
| Johnsen et al.(2009) | Post DBS |
| Kemoun et al.(2003) | Another language |
| Kirchner et al.(2014) | No variables |
| Kleiner et al.(2015) | OFF medication |
| Kleiner et al.(2017) | No variables |
| Kluge et al.(2017) | No variables |
| Kwon et al.(2017) | De novo subjects |
| Knutsson (1972) | No variables |
| Krystkowiak et al.(2006) | Case study |
| Lee et al.(2012) | OFF medication |
| Lewek et al.(2010) | OFF medication |
| Lin & Wagenaar (2018) | No variables |
| Lin et al.(2011) | No variables |
| Lin et al.(2014) | No variables |
| Lin et al.(2016) | No variables |
| Luessi et al.(2012) | No variables |
| Magdalini et al.(2013) | Arabic |
| Mak et al.(2008) | No variables |
| Mancini et al.(2012) | OFF medication |
| Mancini et al.(2017) | OFF medication |
| Maquet et al.(2010) | No variables |
| Maranesi et al.(2015) | No variables |
| Mariani et al.(2013) | No variables |
| Martelli et al.(2017) | No variables |
| Mazzone et al.(2014) | Post DBS |
| McGrath et al. (2019) | No variables |
| McIntosh et al. (1997) | No variables |
| McNeely & Earhart (2012) | No variables |
| McVey et al.(2013) | No variables |
| Mellone et al.(2016) | No variables |
| Melnick et al.(2002) | No full text |
| Memar et al.(2018) | No variables |
| Merello et al.(2010) | OFF medication |
| Mezzarobba et al.(2015) | No variables |
| Mezzarobba et al.(2018) | No variables |
| Mian et al.(2011) | OFF medication |
| Mico-Amigo et al.(2017) | Post DBS |
| Mirek et al.(2003) | Another language |
| Mirek et al.(2007) | Another language |
| Mitoma (1997) | Another language |
| Mizuno et al.(2010) | Another language |
| Mohammadi et al.(2015) | No variables |
| Moreno Catala et al.(2016) | No variables |
| Moreno Izco et al.(2005) | OFF medication |
| Morris et al.(2001) | No full text |
| Morris et al.(1996) | No variables |
| Morris et al.(1996) | No variables |
| Morris et al.(1998) | No variables |
| Murray et al.(1978) | [Parkinsonism](https://www.google.com/search?rlz=1C1GKLC_enBR836BR836&q=parkinsonismo&spell=1&sa=X&ved=0ahUKEwiv3pWbk4fhAhXhILkGHSneALQQkeECCCooAA) |
| Nanhoe-Mahabier et al.(2011) | OFF medication |
| Nanhoe-Mahabier et al.(2013) | OFF medication |
| Nardello et al.(2017) | No variables |
| Nieuwenhuijzen et al.(2006) | No variables |
| Novak & Novak (2006) | Pilot study |
| Otte et al.(2017) | No variables |
| Orcioli-Silva et al.(2018) | Essential Tremor |
| Paquet et al.(2003) | Another language |
| Pedersen et al.(1997) | No full text |
| Pestana et al.(2016) | No variables |
| Pagnussat et al.(2018) | No variables |
| Pasluosta et al.(2018) | No variables |
| Pieruccini-Faria et al.(2014) | No variables |
| Pieruccini-Faria et al.(2016) | No variables |
| Pistacchi et al.(2017) | No variables |
| Rahimi et al.(2013) | No variables |
| Ren et al.(2015) | No variables |
| Robles-Garcia et al.(2015) | OFF medication |
| Rochester et al.(2017) | No variables |
| Rodriguez et al.(2013) | No variables |
| Roemmich et al.(2013) | No variables |
| Rosas et al.(2015) | No variables |
| Rossi & Bacchini (2000) | No variables |
| Rosin et al.(1997) | No variables |
| Rossi et al.(2009) | No variables |
| Salarian et al.(2004) | Post DBS |
| Salarian et al.(2010) | OFF medication |
| Scandalis et al.(2001) | OFF medication |
| Schlachetzki et al.(2017) | No variables |
| Schubert et al.(2005) | No full text |
| Sejdic et al.(2014) | No variables |
| Serrao et al.(2018) | No variables |
| Serrao et al.(2018) | No variables |
| Shah et al.(2018) | No variables |
| Smith et al.(2013) | No variables |
| Shoushtarian et al.(2011) | OFF medication |
| Smpiliris et al.(2013) | No variables |
| Son et al.(2017) | OFF medication |
| Speciali et al.(2012) | No variables |
| Stathis et al.(2012) | No variables |
| Stegemoller et al.(2012) | No variables |
| Stocchi et al.(2015) | OFF medication |
| Suputtitada & Saguanrungsirikul (2012) | No variables |
| Svehlik et al.(2009) | OFF medication |
| Szlufik et al.(2014) | No variables |
| Tan et al.(2011) | No variables |
| Tavakoli et al.(2011) | No variables |
| Terashi et al.(2015) | No variables |
| Thaut et al.(1996) | No control group |
| Tupa et al.(2015) | No variables |
| Ueno et al.(1993) | No variables |
| Vacherot et al.(2010) | OFF medication |
| Vaillancourt et al.(2006) | Post DBS |
| Vallabhajosula et al.(2013) | No variables |
| Van Emmerik et al.(1999) | OFF medication |
| Van Uem et al.(2016) | OFF medication |
| Vercruysse et al.(2012) | OFF medication |
| Vervoort et al.(2015) | OFF medication |
| Vitorio et al.(2013) | No variables |
| Vitorio et al.(2014) | No variables |
| Vysata et al.(2013) | No variables |
| Vitorio et al.(2016) | No variables |
| Volpe et al.(2017) | No control group |
| Von Papen et al.(2014) | No variables |
| Wahid et al.(2015) | No variables |
| Wang et al.(2014) | Another language |
| Warlop et al.(2017) | Pilot study |
| Wells et al.(1999) | OFF medication |
| Wolfsegger et al.(2011) | Another language |
| Xia et al.(2016) | No variables |
| Xu et al.(2018) | Duplicate data |
| Young et al.(2010) | No variables |
| Zampieri et al.(2011) | Pilot study |
| [Zhenlan](https://www.ncbi.nlm.nih.gov/pubmed/?term=Li%20Z%5BAuthor%5D&cauthor=true&cauthor_uid=31515419) et al. (2019) | Protocol study |
| Zijlmans et al.(1996) | OFF medication |

Supplementary material 1.4

Characteristics of the included studies

| **Study** | **Number of participants in PD; and in HC** | **Mean Age PD (years)** | **Mean Age HC (years)** | **H&Y (scores)** | **UPDRS (scores)** | **Disease duration (years)** | **Measurements** | **Device** | **Distance (m)** |
| --- | --- | --- | --- | --- | --- | --- | --- | --- | --- |
| Arias & Cudeiro [35] | PD = 25; HC = 10 | 65.9 ± 7.7 | 65.7 ± 7.7 | 2.5 ± .6 | 53.4 ± 21.3 | 9.0 ± 6.2 | Free Walking | Photocells | 30 |
| Azulay et al. [36] | PD =16; HC =16 | 68.8 ± 4.0 | 67.5 ± 5.0 | 2 to 3 | Not reported | 6.3 | Free Walking | 3D Gait Analysis | 12 |
| Azulay et al. [37] | PD =21; HC =22 | 68.0 ± 11.0 | 67.5 ± 13.9 | 2.4 ± .5 | Not reported | 5.4 ± .7 | Free Walking | 3D Gait Analysis | 12 |
| Bhatt et al. [38] | PD = 10; HC =10 | 72.3 ± 9.8 | 69.6 ± 7.5 | Not reported | 33.4 ± 1.4 | Not reported | Free Walking | 3D Gait Analysis | 6 |
| Blin et al. [39] | PD = 21; HC =58 | 50 to 85 | 60 to 92 | 1 to 4 | Not reported | 1 to 17 | Free Walking | Potentiometer | More than 10 |
| Bond & Morn’s [40] | PD = 12; HC =12 | 65.1 ± 1.3 | 65.3 ± 1.4 | Not reported | Not reported | 9.4 ± 6.5 | Free Walking | 3D Gait Analysis | 15 |
| Brown et al. [41] | PD = 10; HC = 10 | 66.6 ± 6.5 | 65.4 ± 6.3 | 2.3 ± .3 | 28.2 ± 2.4 | 6.4 ± 4.5 | Free Walking | 3D Gait Analysis | 10 |
| Bugalho et al. [42] | PD = 40; HC = 30 | 74.3 ± 6.9 | 73.4 ± 7.1 | 2.2 ± .7 | 17.4 ± 12.3 | 5.8 ± 4.9 | Free Walking | 3D Gait Analysis | 10 |
| Caetano et al. [43] | PD = 8; HC = 8 | 68.7 ± 6.6 | 69.7 ± 4.9 | 1.7 ± .9 | 26.9 ± 13.9 | 4.9 ± 5.5 | Free Walking | 3D Gait Analysis | 5 |
| Carpinella et al. [11] | PD = 7; HC = 7 | 65.9 ± 4.8 | 68.4 ± 2.4 | 1 to 2 | 15.6 ± 3.0 | Not reported | Free Walking | 3D Gait Analysis | 6 |
| Castagna et al. [44] | PD = 15; HC = 15 | 5.7 ± 11.5 | 49.2 ± 1.5 | Not reported | 15.2 ± 1.6 | 14.7 ± 7.1 | Free Walking | 3D Gait Analysis | 6 |
| Chen et al. [45] | PD = 12; HC = 12 | 6.3 ± 6.7 | 56.4 ± 7.0 | 2.3 ± .3 | 2.2 ± 3.0 | 8.0 ± 4.8 | Free Walking | 2D Gait Analysis | 6 |
| Cole et al. [46] | PD = 17; HC = 17 | 66.9 ± 8.7 | 65.1 ± 8.7 | 2.5 ± .8 | 26.6 ± 15.3 | 3.9 ± 2.5 | Free Walking | 3D Gait Analysis | 12 |
| Cole et al. [47] | PD = 31; HC = 53 | 66.5 ± 7.8 | 69.6 ± 8.0 | 1.4 ± .6 | 29.4 ± 1.0 | 4.2 ± 3.3 | Free Walking | 3D Gait Analysis | 9 |
| Danoudis & Iansek [48] | PD = 20; HC = 21 | 68.9 ± 8.8 | 71.7 ± 4.0 | 1 to 5 | 15 to 56 | 5.6 ± 5.5 | Free Walking | Kinetics | 12 |
| De Nunzio et al. [49] | PD = 15; HC = 14 | 68.4 ± 1.9 | 6.2 ± 11.6 | 2.5 ± .6 | 26.8 ± 1.2 | 5.4 ± 4.4 | Free Walking | Kinetics | 10 |
| Del Din et al. [50] | PD = 47; HC = 50 | 69.1 ± 8.3 | 69.8 ± 7.2 | 1 to 3 | 32.0 ± 1.1 | Not reported | Free Walking | Accelerometer | 10 |
| Demonceau et al. [51a] | PD = 32; HC = 32 | 64.5 ± 7.1 | 64.8 ± 9.9 | 1.7 ± .6 | 13.0 ± 6.2 | 1.5 to 5 | Free Walking | Accelerometer | 36 |
| Demonceau et al. [51b] | PD = 32; HC = 32 | 65.3 ± 8.5 | 64.8 ± 9.9 | 2 to 3 | 2.3 ± 8.4 | 8 to 14 | Free Walking | Accelerometer | 36 |
| Dillmann et al. [52a] | PD = 17; HC = 35 | 61.8 ± 9.8 | 6.8 ± 4.7 | 1 to 2 | >20 | Not reported | Treadmill | 3D Gait Analysis | Not reported |
| Dillmann et al. [52b] | PD = 19; HC = 35 | 64.3 ± 8.8 | 6.8 ± 4.7 | 2.5 to 4 | >20 | Not reported | Treadmill | 3D Gait Analysis | Not reported |
| Ebersbach et al. [53] | PD = 30; HC = 30 | 65.0 ± 9.3 | 6.9 ± 8.0 | Not reported | Not reported | Not reported | Free Walking | Kinetics | 10 |
| Egerton et al. [54] | PD = 20; HC = 20 | 68.3 ± 7.9 | 71.8 ± 4.1 | Not reported | Not reported | 6.6 ± 5.8 | Free Walking | Kinetics | 10 |
| Eltoukhy et al. [55] | PD = 8; HC = 11 | 71.0 ± 5.6 | 71.1 ± 7.5 | 1 to 3 | Not reported | Not reported | Free Walking | 3D Gait Analysis | 5 |
| Esser et al. [56] | PD = 14; HC = 10 | 63.4 ± 7.7 | 66.4 ± 4.4 | Not reported | Not reported | 6.1 ± 4.8 | Free Walking | Accelerometer | 10 |
| Esser et al. [57] | PD = 29; HC = 10 | 63.4 ± 7.7 | 66.4 ± 4.4 | Not reported | Not reported | 6.1 ± 4.8 | Free Walking | Accelerometer | 10 |
| Frenkel-Toledo et al. [58] | PD = 36; HC = 30 | 61.2 ± 9.0 | 57.7 ± 7.0 | Not reported | Not reported | Not reported | Free Walking | Kinetics | 35 |
| Frenkel-Toledo et al. [59] | PD = 36; HC = 30 | 61.2 ± 9.0 | 57.7 ± 7.0 | Not reported | Not reported | Not reported | Treadmill | Kinetics | Not reported |
| Galletly & Brauer [60] | PD = 16; HC = 16 | 65.0 ± 9.5 | 65.0 ± 9.6 | Not reported | Not reported | 9.1 ± 4.5 | Free Walking | Accelerometer | 12 |
| Hackney & Earhart [61] | PD = 78; HC = 74 | 65.1 ± 9.5 | 65.0 ± 1.0 | .5 to 3 | 27.5 ± 9.2 | 8.2 ± 5.0 | Free Walking | Kinetics | 5 |
| Hackney & Earhart [62] | PD = 78; HC = 74 | 65.1 ± 9.5 | 65.0 ± 1.0 | .5 to 3 | 27.5 ± 9.2 | 8.2 ± 5.0 | Free Walking | Kinetics | 5 |
| Hausdorff et al. [63] | PD = 29; HC = 26 | 67.2 ± 9.1 | 64.6 ± 6.8 | 2.4 ± .4 | 15.8 ± 4.5 | Not reported | Free Walking | Kinetics | 100 |
| Jaywant et al. [64] | PD = 26; HC = 24 | 65.1 ± 7.9 | 62.5 ± 8.6 | 1 to 3 | 18.6 ± 8.0 | Not reported | Free Walking | Accelerometer | 11 |
| Kimmeskamp & Hennig [65] | PD = 24; HC = 24 | 63.8 ± 1.1 | 66.1 ± 9.2 | Not reported | Not reported | Not reported | Free Walking | Kinetics | 11 |
| Kincses et al. [66] | PD = 40; HC = 49 | 68.0 ± 8.1 | 65.6 ± 5.6 | Not reported | 31.3 ± 13.7 | 6.7 ± 4.5 | Free Walking | 2D Gait Analysis | 4 |
| Latt et al. [67] | PD = 33; HC = 33 | 63.0 ± 4.0 | 67.0 ± 4.0 | 1.0 to 1.0 | 12.0 ± 3.0 | 7.0 ± 2.0 | Free walking | Accelerometer | 20 |
| Lewis et al. [68] | PD = 14; HC = 14 | 71.1 ± 7.6 | 7.5 ± 6.5 | 2.6 ± .8 | Not reported | 9.1 ± 5.7 | Free Walking | 3D Gait Analysis | 10 |
| Lin et al. [69] | PD = 12; HC = 12 | 64.3 ± 8.6 | 51.3 ± 7.4 | 2.5 ± .6 | 26.2 ± 14.1 | Not reported | Free Walking | Kinetics | 4 |
| Lohnes & Earhart [70] | PD = 11; HC = 11 | 7.3 ± 6.8 | 7.8 ± 1.4 | 2 to 3 | 21.6 ± 6.7 | 9.1 ± 5.4 | Free Walking | Kinetics | 5 |
| Lowry et al. [71] | PD = 11; HC = 11 | 68.0 ± 7.7 | 68.9 ± 8.8 | 1.9 ± .8 | Not reported | 5.1 ± 4.1 | Free Walking | Accelerometer | 18 |
| Maggioni et al. [72] | PD = 14; HC = 14 | 67.9 ± 8.1 | 66.6 ± 5.3 | 2.0 ± .6 | 2.4 ± 15.4 | 6.2 ± 4.1 | Free Walking | Kinetics | 10 |
| Mak [73] | PD = 13; HC = 15 | 63.9 ± 7.2 | 61.8 ± 6.0 | 2.4 ± .4 | 22.8 ± 6.1 | 8.0 ± 5.3 | Treadmill | Kinetics | Not reported |
| Mak et al. [74] | PD = 15; HC = 13 | 63.0 ± 4.9 | 6.0 ± 7.1 | 2.1 ± .4 | 14.7 ± 3.8 | 7.7 ± 4.3 | Free Walking | Kinetics | 3.7 |
| McNeely et al. [75] | PD = 22; HC = 20 | 71.3 ± 7.6 | 72.1 ± 6.1 | 2.2 ± .3 | 25.3 ± 6.9 | 7.0 ± 4.2 | Free Walking | Kinetics | 4.8 |
| Morris et al. [8] | PD = 22; HC = 22 | 75.7 ± 6.7 | > 60 | 3.1 ± .7 | Not reported | Not reported | Free Walking | Kinetics | 10 |
| Morris et al. [76] | PD = 15; HC = 15 | 72.2 ± 6.2 | 72.5 ± 6.5 | 2.7 ± .7 | Not reported | Not reported | Free Walking | Kinetics | 12 |
| Morris et al. [77] | PD = 12; HC = 12 | 66.3 ± 9.4 | 50 to 78 | Not reported | 17.8 ± 9.4 | Not reported | Free Walking | 3D Gait Analysis | 10 |
| O'Shea et al. [78] | PD = 15; HC =15 | 68.3 ± 6.6 | 67.7 ± 7.0 | Not reported | Not reported | Not reported | Free Walking | 2D Gait Analysis | 14 |
| Peppe et al. [24] | PD = 16; HC = 13 | 66.5 ± 9.8 | 63.2 ± 11.2 | 2.3 ± .5 | 31.3 ± 1.0 | 6.7 ± 4.2 | Free Walking | 3D Gait Analysis | 8 |
| Pieruccini-Faria et al. [79] | PD = 12; HC = 12 | 67.0 ± 6.2 | Not reported | 2.1 ± .6 | 26.7 ± 18.0 | 7.1 ± 5.5 | Free Walking | 3D Gait Analysis | 8 |
| Rabin et al. [80] | PD = 16; HC = 16 | 71.0 ± 9.6 | 50 to 78 | 2.0 ± .5 | 3.5 ± 9.0 | 8.4 ± 5.5 | Free Walking | 3D Gait Analysis | 6 |
| Rafferty et al. [81] | PD = 24; HC = 23 | 59.0 ± 4.6 | 61.2 ± 7.7 | Not reported | Not reported | Not reported | Free Walking | Kinetics | 10 |
| Rochester et al. [82] | PD = 22; HC = 22 | 7.2 ± 9.7 | 67.4 ± 8.4 | 1 to 3 | 29.1 ± 9.5 | 1.8 ± .1 | Free Walking | 3D Gait Analysis | 7 |
| Roiz et al. [83] | PD = 12; HC = 15 | 63.7 ± 8.3 | 59.1 ± 4.2 | 2.8 ± .5 | Not reported | 6.6 ± 4.3 | Free Walking | 3D Gait Analysis | 10 |
| Salazar et al. [84] | PD = 19; HC = 13 | 66.3 ± 5.6 | 63.2 ± 4.5 | 1 to 3 | 2.6 ± 1.1 | 4.9 ± 4.2 | Free Walking | 3D Gait Analysis | 1.4 |
| Santos et al. [85a] | PD = 10; HC = 10 | 67.0 ± 5.2 | 67.5 ± 6.5 | 2.0 ± .2 | 31.8 ± 6.9 | 4.6 ± 1.6 | Free Walking | 3D Gait Analysis | 8 |
| Santos et al. [85B] | PD = 10; HC = 10 | 71.7 ± 5.0 | 71.4 ± 6.4 | 1.8 ± .2 | 29.1 ± 6.7 | 3.5 ± .8 | Free Walking | 3D Gait Analysis | 8 |
| Sofuwa et al. [86] | PD = 15; HC = 9 | 63.1 ± 8.4 | 64.4 ± 4.6 | 2.6 ± .6 | 16.1 ± 6.4 | 11.3 ± 3.8 | Free Walking | 3D Gait Analysis | 8 |
| Stolze et al. [87] | PD = 10; HC = 12 | 66.4 ± 6.7 | 74.6 ± 5.9 | 2.7 ± .4 | 29.6 ± 16.0 | 7.7 ± 4.8 | Free Walking | 3D Gait Analysis | 13 |
| Tramonti et al. [88] | PD = 10; HC = 10 | 73.2 ± 8.1 | 68.8 ± 1.0 | 2.8 ± 1.0 | 26.1 ± 12.4 | Not reported | Free Walking | 3D Gait Analysis | 10 |
| Trojaniello et al. [89] | PD = 10; HC = 10 | 73.8 ± 5.7 | 69.7 ± 5.8 | Not reported | Not reported | Not reported | Free Walking | Kinetics | 12 |
| Turcato et al. [90] | PD = 18; HC = 18 | 71.4 ± 8.0 | 72.7 ± 7.6 | 2.1 ± 1.8 | 9 to 27 | 8.6 ± 3.1 | Free Walking | Kinetics | 20 |
| Van Wegen et al. [91] | PD = 13; HC = 7 | 62.3 ± 9.8 | 59.2 ± 1.2 | 2.3 ± .5 | 52.9 ± 11.1 | 5.5 ± 3.5 | Free Walking | 3D Gait Analysis | 10 |
| Vaugoyeau et al. [92] | PD = 10; HC = 5 | 62.2 ± 5.5 | 61.8 ± 5.4 | 3.3 ± .5 | 27.8 ± 5.4 | 13.2 ± 6.9 | Free Walking | Kinetics | 10 |
| Vieregge et al. [93] | PD = 17; HC = 33 | 68.8 ± 7.4 | 69.9 ± 7.0 | 2 to 3 | 37.5 ± 16.8 | Not reported | Free walking | Kinetics | 13 |
| Vitório et al. [102] | PD = 12; HC = 12 | 67.0 ± 6.2 | 67.0 ± 6.4 | 2.1 ± .6 | 3.9 ± 19.3 | 7.1 ± 5.5 | Free Walking | 3D Gait Analysis | 8 |
| Vitório et al. [95] | PD = 12; HC = 12 | 69.8 ± 5.7 | 69.6 ± 6.0 | 1.4 ± .5 | 19.8 ± 12.2 | Not reported | Free Walking | 3D Gait Analysis | 8 |
| Vitório et al. [94] | PD = 19; HC = 15 | 64.8 ± 9.3 | 66.8 ± 7.7 | Not reported | 24.3 ± 8.5 | Not reported | Free Walking | 3D Gait Analysis | 8 |
| Wahid et al. [96] | PD = 28; HC = 29 | 68.5 ± 6.6 | 69.1 ± 6.4 | 2.5 | Not reported | Not reported | Free Walking | 3D Gait Analysis | 10 |
| Willems et al. [97] | PD = 10; HC = 10 | 6.6 ± 6.2 | 63.6 ± 5.1 | 2.7 ± .6 | 24.7 ± 12.6 | 6.2 ± 3.0 | Free Walking | 3D Gait Analysis | 8 |
| Xu et al. [98] | PD = 9; HC = 9 | 67.7 ± 7.1 | 67.7 ± 8.0 | 2.4 ± .3 | 36.1 ± 11.8 | Not reported | Free Walking | 3D Gait Analysis | 7.3 |
| Yang et al. [99] | PD = 18; HC = 17 | 68.6 ± 11.1 | 68.9 ± 7.0 | 1 to 2 | Not reported | Not reported | Free Walking | Kinetics | 10 |
| Zhang et al. [103] | PD = 15; HC = 11 | 63.7 ± 5.6 | 65.2 ± 4.0 | 2.8 ± .4 | 1.9 ± 6.4 | 8.0 ± 3.0 | Free Walking | 3D Gait Analysis | 5 |
| Zhou et al. [100] | PD = 12; HC = 12 | 61.6 ± 11.7 | 68.0 ± 6.4 | 1 to 3 | 11.0 ± 5.4 | 6.7 ± 3.9 | Free Walking | 3D Gait Analysis | 5 |
| Zijlstra et al. [101] | PD = 10; HC = 8 | 44 to 74 | 55 to 60 | 1.5 to 3 | Not reported | Not reported | Free Walking | 3D Gait Analysis | 10 |

Supplementary material 1.5

Methodological Quality of the Included Trials.

| **Studies** | **Quality Index item Number** | | | | | | | | | | | | |  | **Percentage score**  **(100%)** | **Quality category** |
| --- | --- | --- | --- | --- | --- | --- | --- | --- | --- | --- | --- | --- | --- | --- | --- | --- |
|  | **1** | **2** | **3** | **5** | **6** | **7** | **10** | **11** | **12** | **18** | **20** | **21** | **22** | **Total** |  |  |
| Arias & Cudeiro [35] | 1 | 1 | 1 | 2 | 1 | 1 | 1 | 1 | 1 | 1 | 1 | 1 | 1 | 14 | 100 | High |
| Azulay et al. [36] | 1 | 1 | 0 | 2 | 1 | 1 | 1 | 1 | 1 | 1 | 1 | 1 | 0 | 12 | 86 | High |
| Azulay et al. [37] | 1 | 1 | 0 | 2 | 1 | 1 | 1 | 0 | 0 | 1 | 1 | 1 | 0 | 10 | 71 | High |
| Bhatt et al. [38] | 1 | 1 | 0 | 2 | 1 | 1 | 1 | 1 | 1 | 1 | 1 | 1 | 0 | 12 | 86 | High |
| Blin et al. [39] | 1 | 1 | 0 | 1 | 1 | 1 | 1 | 0 | 0 | 1 | 1 | 1 | 0 | 9 | 64 | Medium |
| Bond & Morn’s [40] | 1 | 1 | 0 | 2 | 1 | 1 | 1 | 1 | 1 | 1 | 1 | 1 | 0 | 12 | 86 | High |
| Brown et al. [41] | 1 | 1 | 1 | 2 | 1 | 1 | 1 | 1 | 1 | 1 | 1 | 1 | 0 | 13 | 93 | High |
| Bugalho et al. [42] | 1 | 1 | 1 | 2 | 1 | 1 | 1 | 1 | 1 | 1 | 1 | 1 | 1 | 14 | 100 | High |
| Caetano et al. [43] | 1 | 1 | 1 | 2 | 1 | 1 | 1 | 1 | 1 | 1 | 1 | 1 | 0 | 13 | 93 | High |
| Carpinella et al. [11] | 1 | 1 | 0 | 1 | 1 | 1 | 1 | 1 | 1 | 1 | 1 | 1 | 0 | 11 | 79 | High |
| Castagna et al. [44] | 1 | 0 | 0 | 2 | 1 | 1 | 1 | 0 | 0 | 1 | 1 | 1 | 0 | 9 | 64 | Medium |
| Chen et al. [45] | 1 | 1 | 1 | 1 | 1 | 1 | 0 | 1 | 1 | 1 | 1 | 1 | 0 | 11 | 79 | High |
| Cole et al. [46] | 1 | 1 | 1 | 2 | 1 | 1 | 1 | 1 | 1 | 1 | 1 | 1 | 1 | 14 | 100 | High |
| Cole et al. [47] | 1 | 1 | 1 | 2 | 1 | 1 | 1 | 1 | 1 | 1 | 1 | 1 | 1 | 14 | 100 | High |
| Danoudis & Iansek [48] | 1 | 1 | 1 | 2 | 1 | 1 | 1 | 1 | 1 | 1 | 1 | 1 | 0 | 13 | 93 | High |
| De Nunzio et al. [49] | 1 | 1 | 1 | 2 | 1 | 1 | 1 | 0 | 0 | 1 | 1 | 1 | 0 | 11 | 79 | High |
| Del Din et al. [50] | 1 | 1 | 1 | 0 | 1 | 1 | 1 | 1 | 1 | 1 | 1 | 1 | 1 | 12 | 86 | High |
| Demonceau et al. [51] | 1 | 1 | 1 | 2 | 1 | 1 | 1 | 1 | 1 | 1 | 1 | 1 | 0 | 13 | 93 | High |
| Dillmann et al. [52] | 1 | 1 | 1 | 2 | 1 | 1 | 1 | 1 | 1 | 1 | 1 | 1 | 0 | 13 | 93 | High |
| Ebersbach et al. [53] | 1 | 1 | 0 | 2 | 1 | 1 | 1 | 1 | 1 | 1 | 1 | 1 | 0 | 12 | 86 | High |
| Egerton et al. [54] | 1 | 1 | 0 | 2 | 1 | 1 | 1 | 1 | 1 | 1 | 1 | 1 | 0 | 12 | 86 | High |
| Eltoukhy et al. [55] | 1 | 1 | 0 | 2 | 1 | 1 | 1 | 1 | 1 | 1 | 1 | 1 | 0 | 12 | 86 | High |
| Esser et al. [56] | 1 | 1 | 0 | 2 | 1 | 1 | 1 | 0 | 0 | 1 | 1 | 1 | 0 | 10 | 71 | High |
| Esser et al. [57] | 1 | 1 | 0 | 2 | 1 | 1 | 1 | 1 | 1 | 1 | 1 | 1 | 0 | 12 | 86 | High |
| Frenkel-Toledo et al. [58] | 1 | 1 | 0 | 2 | 1 | 1 | 1 | 0 | 0 | 1 | 1 | 1 | 0 | 10 | 71 | High |
| Frenkel-Toledo et al. [59] | 1 | 1 | 0 | 2 | 1 | 1 | 1 | 0 | 0 | 1 | 1 | 1 | 0 | 10 | 71 | High |
| Galletly & Brauer [60] | 1 | 1 | 0 | 1 | 1 | 1 | 1 | 0 | 0 | 0 | 1 | 1 | 0 | 8 | 57 | Medium |
| Hackney & Earhart [61] | 1 | 1 | 1 | 2 | 1 | 1 | 1 | 1 | 1 | 1 | 1 | 1 | 0 | 13 | 93 | High |
| Hackney & Earhart [62] | 1 | 1 | 1 | 2 | 1 | 1 | 1 | 1 | 1 | 1 | 1 | 1 | 0 | 13 | 93 | High |
| Hausdorff et al. [63] | 1 | 1 | 1 | 2 | 1 | 1 | 1 | 1 | 1 | 1 | 1 | 1 | 0 | 13 | 93 | High |
| Jaywant et al. [64] | 1 | 1 | 1 | 2 | 1 | 1 | 1 | 1 | 1 | 1 | 1 | 1 | 0 | 13 | 93 | High |
| Kimmeskamp & Hennig [65] | 1 | 1 | 0 | 2 | 1 | 1 | 1 | 1 | 1 | 1 | 1 | 1 | 0 | 12 | 86 | High |
| Kincses et al. [66] | 1 | 1 | 1 | 2 | 1 | 1 | 0 | 1 | 1 | 1 | 1 | 1 | 0 | 12 | 86 | High |
| Latt et al. [67] | 1 | 1 | 1 | 2 | 1 | 1 | 0 | 0 | 0 | 1 | 1 | 1 | 0 | 10 | 71 | High |
| Lewis et al. [68] | 1 | 1 | 0 | 2 | 1 | 1 | 1 | 1 | 1 | 1 | 1 | 1 | 0 | 12 | 86 | High |
| Lin et al. [69] | 1 | 1 | 1 | 2 | 1 | 1 | 0 | 1 | 1 | 1 | 1 | 1 | 0 | 12 | 86 | High |
| Lohnes & Earhart [70] | 1 | 1 | 1 | 2 | 1 | 1 | 1 | 0 | 0 | 1 | 1 | 1 | 0 | 11 | 79 | High |
| Lowry et al. [71] | 1 | 1 | 0 | 2 | 1 | 1 | 1 | 1 | 1 | 1 | 1 | 1 | 0 | 12 | 86 | High |
| Maggioni et al. [72] | 1 | 1 | 1 | 2 | 1 | 1 | 1 | 1 | 1 | 1 | 1 | 1 | 0 | 13 | 93 | High |
| Mak [73] | 1 | 1 | 1 | 2 | 1 | 1 | 1 | 0 | 0 | 1 | 1 | 1 | 0 | 11 | 79 | High |
| Mak et al. [74] | 1 | 1 | 1 | 2 | 1 | 1 | 1 | 1 | 1 | 1 | 1 | 1 | 0 | 13 | 93 | High |
| McNeely et al. [75] | 1 | 1 | 1 | 2 | 1 | 1 | 1 | 1 | 1 | 1 | 1 | 1 | 0 | 13 | 93 | High |
| Morris et al. [8] | 1 | 1 | 0 | 1 | 1 | 1 | 1 | 1 | 1 | 0 | 1 | 1 | 0 | 10 | 71 | High |
| Morris et al. [76] | 1 | 1 | 0 | 2 | 1 | 1 | 1 | 0 | 0 | 0 | 1 | 1 | 0 | 9 | 64 | Medium |
| Morris et al. [77] | 1 | 1 | 0 | 2 | 1 | 1 | 1 | 1 | 1 | 1 | 1 | 1 | 0 | 12 | 86 | High |
| O'Shea et al. [78] | 1 | 1 | 0 | 1 | 1 | 1 | 0 | 0 | 0 | 1 | 1 | 1 | 0 | 8 | 57 | Medium |
| Peppe et al. [24] | 1 | 1 | 1 | 2 | 1 | 1 | 0 | 0 | 0 | 1 | 1 | 1 | 0 | 10 | 71 | High |
| Pieruccini-Faria et al. [79] | 1 | 1 | 1 | 2 | 1 | 1 | 1 | 1 | 1 | 1 | 1 | 1 | 0 | 13 | 93 | High |
| Rabin et al. [80] | 1 | 1 | 1 | 2 | 1 | 1 | 1 | 1 | 1 | 1 | 1 | 1 | 0 | 13 | 93 | High |
| Rafferty et al. [81] | 1 | 1 | 0 | 1 | 1 | 1 | 1 | 1 | 1 | 1 | 1 | 1 | 1 | 12 | 86 | High |
| Rochester et al. [82] | 1 | 1 | 1 | 2 | 1 | 1 | 1 | 1 | 1 | 1 | 1 | 1 | 0 | 13 | 93 | High |
| Roiz et al. [83] | 1 | 1 | 0 | 1 | 1 | 1 | 1 | 0 | 0 | 1 | 1 | 1 | 0 | 9 | 64 | Medium |
| Salazar et al. [84] | 1 | 1 | 1 | 2 | 1 | 1 | 1 | 1 | 1 | 1 | 1 | 1 | 0 | 13 | 93 | High |
| Santos et al. [85] | 1 | 1 | 1 | 2 | 1 | 1 | 1 | 1 | 1 | 1 | 1 | 1 | 0 | 13 | 93 | High |
| Sofuwa et al. [86] | 1 | 1 | 1 | 2 | 1 | 1 | 1 | 1 | 1 | 1 | 1 | 1 | 0 | 13 | 93 | High |
| Stolze et al. [87] | 1 | 1 | 1 | 2 | 1 | 1 | 0 | 1 | 1 | 1 | 1 | 1 | 0 | 12 | 86 | High |
| Tramonti et al. [88] | 1 | 1 | 1 | 2 | 1 | 1 | 1 | 1 | 1 | 1 | 1 | 1 | 0 | 13 | 93 | High |
| Trojaniello et al. [89] | 1 | 1 | 0 | 1 | 1 | 1 | 1 | 0 | 0 | 1 | 1 | 1 | 0 | 9 | 64 | Medium |
| Turcato et al. [90] | 1 | 1 | 1 | 2 | 1 | 1 | 1 | 1 | 1 | 1 | 1 | 1 | 0 | 13 | 93 | High |
| Van Wegen et al. [91] | 1 | 1 | 1 | 2 | 1 | 1 | 0 | 1 | 1 | 1 | 1 | 1 | 0 | 12 | 86 | High |
| Vaugoyeau et al. [92] | 1 | 1 | 1 | 2 | 1 | 1 | 1 | 1 | 1 | 1 | 1 | 1 | 0 | 13 | 93 | High |
| Vieregge et al. [93] | 1 | 1 | 1 | 2 | 1 | 1 | 0 | 1 | 1 | 1 | 1 | 1 | 0 | 12 | 86 | High |
| Vitório et al. [102] | 1 | 1 | 1 | 2 | 1 | 1 | 1 | 1 | 1 | 1 | 1 | 1 | 0 | 13 | 93 | High |
| Vitório et al. [95] | 1 | 1 | 1 | 1 | 1 | 1 | 1 | 1 | 1 | 1 | 1 | 1 | 0 | 12 | 86 | High |
| Vitório et al. [94] | 1 | 1 | 0 | 1 | 1 | 1 | 1 | 1 | 1 | 1 | 1 | 1 | 0 | 11 | 79 | High |
| Wahid et al. [96] | 1 | 1 | 0 | 1 | 1 | 1 | 1 | 0 | 0 | 1 | 1 | 1 | 0 | 9 | 64 | Medium |
| Willems et al. [97] | 1 | 1 | 1 | 2 | 1 | 1 | 0 | 1 | 1 | 1 | 1 | 1 | 0 | 12 | 86 | High |
| Xu et al. [98] | 1 | 1 | 1 | 1 | 1 | 1 | 1 | 1 | 1 | 0 | 1 | 1 | 0 | 11 | 79 | High |
| Yang et al. [99] | 1 | 1 | 0 | 2 | 1 | 1 | 1 | 1 | 1 | 1 | 1 | 1 | 0 | 12 | 86 | High |
| Zhang et al. [103] | 1 | 1 | 1 | 2 | 1 | 1 | 0 | 0 | 0 | 1 | 1 | 1 | 0 | 10 | 71 | High |
| Zhou et al. [100] | 1 | 0 | 1 | 1 | 1 | 1 | 1 | 1 | 1 | 1 | 1 | 1 | 0 | 11 | 79 | High |
| Zijlstra et al. [101] | 1 | 1 | 0 | 2 | 1 | 1 | 0 | 1 | 1 | 0 | 1 | 1 | 0 | 10 | 71 | High |
|  |  |  |  |  |  |  |  |  |  |  |  |  |  |  |  |  |
