# Supplementary figures and images for: Gait parameters of Parkinson’s disease compared with healthy controls: a systematic review and meta-analysis
Source: Sci Rep. 2021 Jan 12;11:752. doi: 10.1038/s41598-020-80768-2 (PMC7804291; doi:10.1038/s41598-020-80768-2)

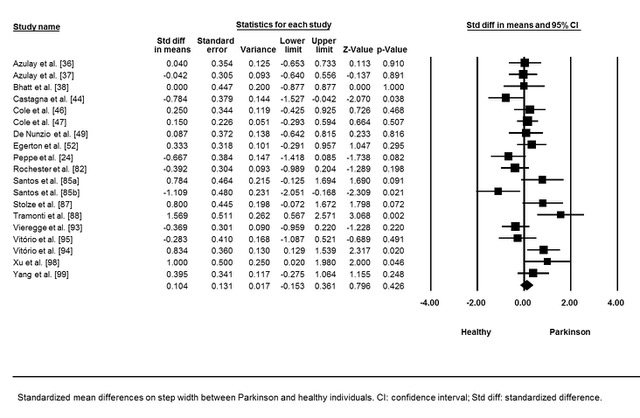

Supplement: Supplementary file 2 — Supplementary Information 1.6. [file 41598_2020_80768_MOESM2_ESM.tiff]

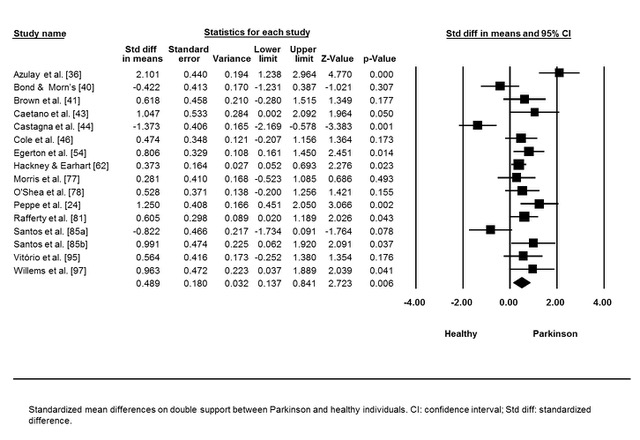

Supplement: Supplementary file 3 — Supplementary Information 1.7. [file 41598_2020_80768_MOESM3_ESM.tiff]

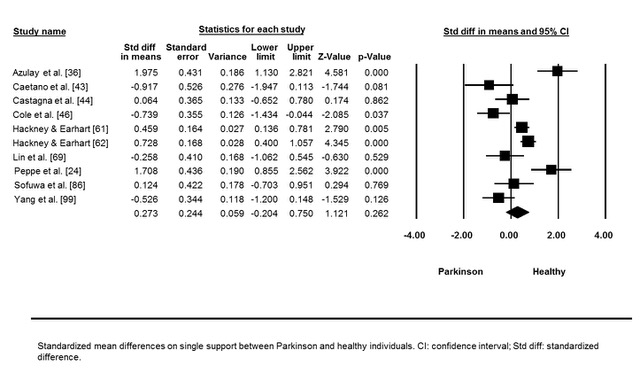

Supplement: Supplementary file 4 — Supplementary Information 1.8. [file 41598_2020_80768_MOESM4_ESM.tiff]

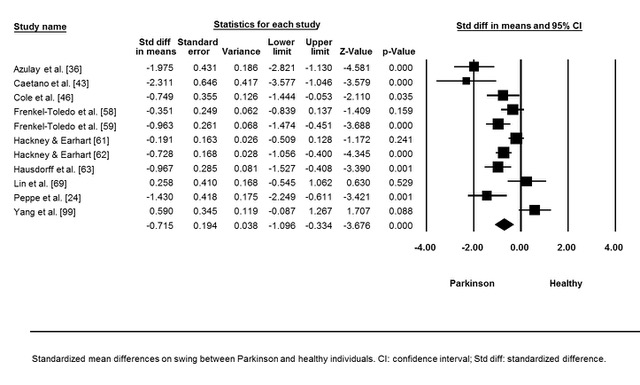

Supplement: Supplementary file 5 — Supplementary Information 1.9. [file 41598_2020_80768_MOESM5_ESM.tiff]

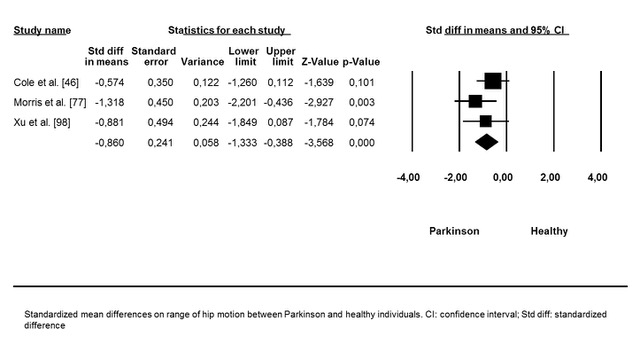

Supplement: Supplementary file 6 — Supplementary Information 2.0. [file 41598_2020_80768_MOESM6_ESM.tiff]

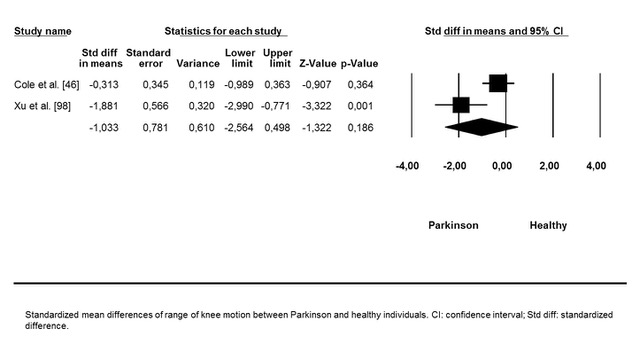

Supplement: Supplementary file 7 — Supplementary Information 2.1. [file 41598_2020_80768_MOESM7_ESM.tiff]

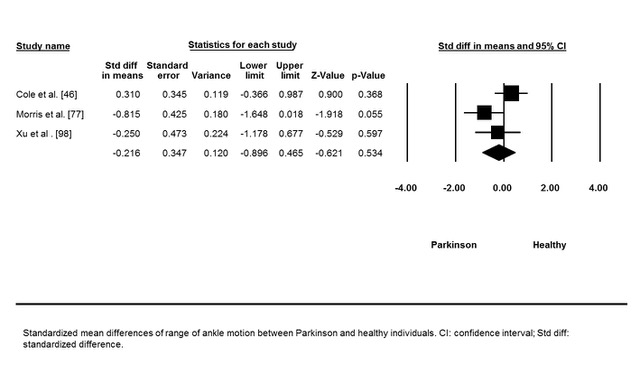

Supplement: Supplementary file 8 — Supplementary Information 2.2. [file 41598_2020_80768_MOESM8_ESM.tiff]

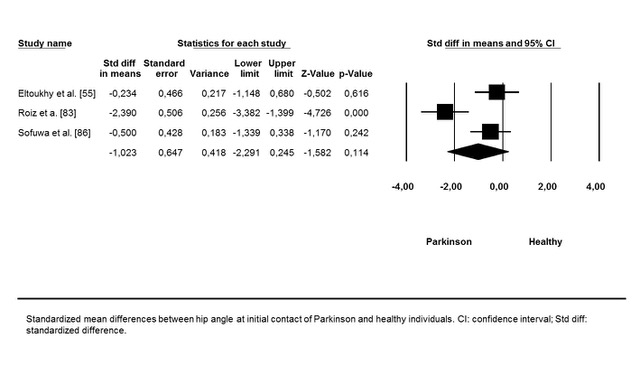

Supplement: Supplementary file 9 — Supplementary Information 2.3. [file 41598_2020_80768_MOESM9_ESM.tiff]

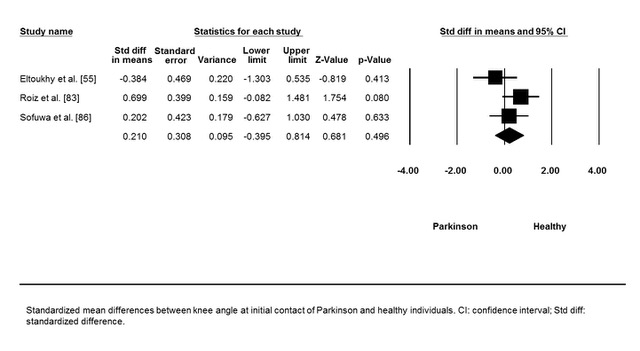

Supplement: Supplementary file 10 — Supplementary Information 2.4. [file 41598_2020_80768_MOESM10_ESM.tiff]

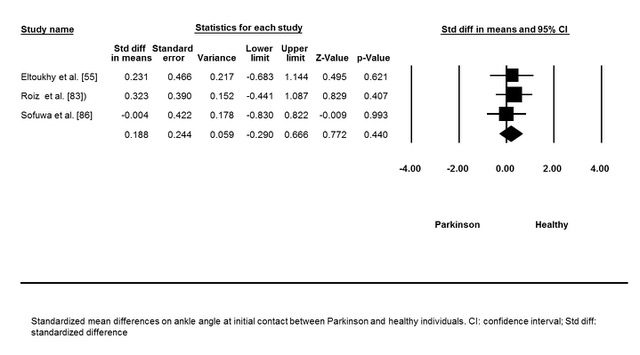

Supplement: Supplementary file 11 — Supplementary Information 2.5. [file 41598_2020_80768_MOESM11_ESM.tiff]
