# Supplementary material for: Gait parameters of Parkinson’s disease compared with healthy controls: a systematic review and meta-analysis
Source: Sci Rep. 2021 Jan 12;11:752. doi: 10.1038/s41598-020-80768-2 (PMC7804291; doi:10.1038/s41598-020-80768-2)
Supplement: Supplementary file 12 — Supplementary Information 2.6. [file 41598_2020_80768_MOESM12_ESM.docx]

Supplementary material 2.6

Mean and standard deviation of gait variable of included studies.

| Variables | Parkinson Group (mean±sd) | Control Group  (mean±sd) |
| --- | --- | --- |
| Speed (m.s^-1^) | 1.11±.44 | 1.24±.37 |
| Stride length (m) | 1.23±.20 | 1.37±.15 |
| Cadence (step.min^-1^) | 101.90±1.56 | 101.23±9.37 |
| Step width (m) | .11±.06 | .09±.06 |
| Double support (%) | 22.10±4 | 20.58±4.98 |
| Single support (%) | 68.39±2.44 | 67.47±3.33 |
| Swing support (%) | 36.07±2.84 | 38.75±8.31 |
| ROM Hip (degree) | 39.39±6.38 | 45.08±5.32 |
| ROM Knee (degree) | 55.90±5.11 | 61.59±4.86 |
| ROM Ankle (degree) | 25.07±4.16 | 26.20±4.41 |
| ROM Hip (initial contact, degree) | 25.22±7.50 | 32.22±5.60 |
| ROM Knee (initial contact, degree) | 8.32±5.21 | 7.14±4.63 |
| ROM Ankle (initial contact, degree) | 1.89±2.71 | 1.40±2.08 |

**NOTE:** sd - standard deviation; ROM - range of motion.
